# Passive Immunization with Phospho-Tau Antibodies Reduces Tau Pathology and Functional Deficits in Two Distinct Mouse Tauopathy Models

**DOI:** 10.1371/journal.pone.0125614

**Published:** 2015-05-01

**Authors:** Sethu Sankaranarayanan, Donna M. Barten, Laurel Vana, Nino Devidze, Ling Yang, Gregory Cadelina, Nina Hoque, Lynn DeCarr, Stefanie Keenan, Alan Lin, Yang Cao, Bradley Snyder, Bin Zhang, Magdalena Nitla, Gregg Hirschfeld, Nestor Barrezueta, Craig Polson, Paul Wes, Vangipuram S. Rangan, Angela Cacace, Charles F. Albright, Jere Meredith, John Q. Trojanowski, Virginia M-Y. Lee, Kurt R. Brunden, Michael Ahlijanian

**Affiliations:** 1 Research and Development, Bristol-Myers Squibb, Wallingford, Connecticut, United States of America; 2 Center for Neurodegenerative Disease Research, Perelman School of Medicine, University of Pennsylvania, Philadelphia, Pennsylvania, United States of America; 3 Research and Development, Bristol-Myers Squibb, Redwood City, California, United States of America; Inserm U837, FRANCE

## Abstract

In Alzheimer’s disease (AD), an extensive accumulation of extracellular amyloid plaques and intraneuronal tau tangles, along with neuronal loss, is evident in distinct brain regions. Staging of tau pathology by postmortem analysis of AD subjects suggests a sequence of initiation and subsequent spread of neurofibrillary tau tangles along defined brain anatomical pathways. Further, the severity of cognitive deficits correlates with the degree and extent of tau pathology. In this study, we demonstrate that phospho-tau (p-tau) antibodies, PHF6 and PHF13, can prevent the induction of tau pathology in primary neuron cultures. The impact of passive immunotherapy on the formation and spread of tau pathology, as well as functional deficits, was subsequently evaluated with these antibodies in two distinct transgenic mouse tauopathy models. The rTg4510 transgenic mouse is characterized by inducible over-expression of P301L mutant tau, and exhibits robust age-dependent brain tau pathology. Systemic treatment with PHF6 and PHF13 from 3 to 6 months of age led to a significant decline in brain and CSF p-tau levels. In a second model, injection of preformed tau fibrils (PFFs) comprised of recombinant tau protein encompassing the microtubule-repeat domains into the cortex and hippocampus of young P301S mutant tau over-expressing mice (PS19) led to robust tau pathology on the ipsilateral side with evidence of spread to distant sites, including the contralateral hippocampus and bilateral entorhinal cortex 4 weeks post-injection. Systemic treatment with PHF13 led to a significant decline in the spread of tau pathology in this model. The reduction in tau species after p-tau antibody treatment was associated with an improvement in novel-object recognition memory test in both models. These studies provide evidence supporting the use of tau immunotherapy as a potential treatment option for AD and other tauopathies.

## Introduction

Alzheimer’s disease (AD) is a devastating and costly disease accounting for 50–80% of senile dementia cases. Worldwide, over 35 million people suffer from dementia and the number is projected to double in the next 20 years [1–2]. In AD, a progressive development of extracellular amyloid aggregates and intracellular tau-containing neurofibrillary tangles (NFTs) is evident, along with neuronal loss and memory dysfunction. [3–5]. Seminal studies based on post-mortem staging of tau pathology in AD subjects suggested that tau pathology spreads along distinct brain anatomical pathways, and the severity of cognitive deficits appears to correlate with the amount and extent of NFT pathology [6–8]. These studies and others have led to the hypothesis that the spread of tau pathology may be due to transmission of toxic tau species along synaptically connected brain regions, and a number of recent studies provide support for this model of pathological tau spreading [9, 10]. In fact, this model is consistent with a common theme of transmission of protein-specific pathologies in a variety of neurodegenerative disorders, including other tauopathies, Parkinsons’s disease, amyotrophic lateral sclerosis, Huntington’s disease and other prion diseases [11–13].

While the nature of the transmissible tau species is unknown, extensive work is ongoing to develop in vitro and in vivo models to understand the underlying nature and mechanism of this cell-to-cell tau spreading [14, 15]. In a variety of cellular systems, including primary neurons, treatment with exogenously prepared tau fibrils made from recombinant proteins or extracts from diseased brains resulted in an increase in detergent insoluble tau and phospho-tau (p-tau) levels [16, 17]. Although cell-to-cell spread of tau pathology is evident in cellular systems overexpressing mutant tau, these are very low frequency events, possibly due to lack of synaptic recycling machinery and cellular connectivity in these in vitro systems [18, 19]. However, robust induction and spread of tau pathology is evident in transgenic mice expressing P301S mutant tau (PS19 mice) following intraparenchymal injection of pre-formed fibrils (PFFs) prepared from recombinant P301L mutant form of tau comprised of only the four microtubule binding repeats of tau (K18PL), or from full-length P301L tau [10, 20, 21]. A similar but less robust tau pathology and spread was evident in wild-type (WT) or tau transgenic mice injected with brain extracts from human AD or tauopathy subjects [9, 22]. These studies demonstrated that tau pathology can be induced not only at the site of misfolded tau injection, but also in synaptically connected brain regions, suggestive of transmission of tau pathology in vivo. In fact, in a tau transgenic mouse designed to express mutant P301L tau specifically in the entorhinal cortex, an age-dependent spread of tau pathology was observed in first-order, synaptically connected dentate granule cells in the hippocampus, providing evidence for direct synaptic spread of tau pathology [23, 24]. Taken together, these results suggest that trans-neuronal uptake and spread of a transmissible tau species may drive pathology, leading to synaptic and cognitive deficits in human AD and related tauopathies.

Even prior to the development of tau transmission models, many groups have tried to define ways to target and reduce pathology in tauopathy models. Active immunization with peptides comprised of specific regions of the tau protein, or passive immunization using monoclonal antibodies directed to a variety of p-tau, conformational or linear tau epitopes, has resulted in reductions in tau pathology in tau over-expression aging models [25–30], with some studies showing functional improvement in motor [25, 28, 31] or cognitive measures [27, 30]. While these studies suggest a potential role for targeting tau with antibodies in AD and other tauopathies, it remains unclear if the age-dependent development of pathology in the employed transgenic models is due a transmissible spread of pathologic tau and/or to different rates of cell autonomous development of pathology. Moreover, the mechanism(s) by which antibodies have reduced tau pathology in these studies is not fully understood; i.e., immunization could result in a reduction of transmission, increased cellular clearance or both [32, 33]. Notably, no passive or active immunization efficacy studies have directly addressed the impact on transmission of pathological tau species.

Here, we have evaluated two distinct p-tau antibodies targeting the pT231 (PHF6) and pS396 (PHF13) sites for their in vitro binding properties, as well as for their ability to prevent the induction of tau pathology in primary neuron cultures and in two different mouse models of tauopathy. In both the rTg4510 inducible tau over-expression mouse model and in a model of tau pathology induction and spread after tau PFF injection into the brain parenchyma of PS19 mice, treatment with p-tau-targeted antibodies led to a significant decline in tau species and improved functional performance in a novel-object recognition test. We have also determined the impact of antibody treatment on CSF tau and p-tau levels, and have assessed the systemic antibody exposures that mediate these in vivo effects. These data provide important evidence of the efficacy of tau immunotherapy in both over-expression and transmission models of tauopathy in mice.

## Materials and Methods

### Antibody affinity measurements

Antibody affinities were measured using the Octet Red system (Fortebio-Pall, CA), which uses Bio-Layer Interferometry for real-time analysis of antibody-antigen interactions. Octet red streptavidin probes (Cat # 18–5019, Fortebio-Pall, CA) loaded with biotinylated phospho-peptides was used as the capture peptide for PHF13 (anti-pS396 tau) and PHF6 (anti-pT231) antibodies, respectively. Biotinylated pS396 peptide (Biotin-HGAEIVYK-pS-PVVSGDT, aa 388–403) and biotinylated pT231 peptide (Biotin-TREPKKVAVVR-pT-PPKSPSSAKSR, aa 220–240) were used as the capture peptides, respectively. Probes were washed, followed by antibody binding and washout to monitor association and dissociation kinetics over a range of antibody concentrations. The different steps included, probe equilibration (50 s), peptide loading (200–300 s), baseline signal following a wash step (100 s), antibody association (600 s) and dissociation (600 s), respectively. Data were processed after baseline subtraction within each probe and isotype control antibody as a reference signal to determine association and dissociation kinetics and derive the binding affinity constant (K_D_) of respective antibodies.

### Effect of antibody immunodepletion on tau pathology in primary neuronal cultures induced with rTg4510 mouse brain homogenates

Rat primary neurons derived from embryonic day 18 pups and were cultured in Minimum-Essential Medium with B27 supplement in 96-well poly-d-lysine coated clear bottom plates (Corning, NY). Neurons were infected with adeno-associated virus carrying P301L T40 (2N4R) tau (Genedetect, FL) at 4 days in-vitro (DIV). DIV 7 neurons were then treated with either 100 nM K18PL tau PFFs or with soluble brain extracts from 8 month old rTg4510 mice at 3000X dilution (equivalent of 1–5 nM total tau). Brain extracts were prepared as described in the methods section on brain extraction. Negative controls had just media additions without PFFs. For immunodepletion experiments, rTg4510 brain extracts were incubated overnight with 30 nM of respective anti-tau antibodies coupled to protein G beads. At DIV14, neurons were fixed with 4% paraformaldehyde in the presence of 0.1% TritonX100, to isolate detergent-insoluble tau aggregates. Fixed neurons were then stained for tau using the conformational MC1 antibody (mouse monoclonal kindly provided by Dr. Peter Davies), and β3-tubulin rabbit polyclonal antibody (Covance). Neurons were counterstained with Hoechst nuclear marker, and Alexa 488-labeled anti-rabbit and Alexa 555-labeled anti-mouse antibodies (Invitrogen, CA). Plates were imaged using the Arrayscan system (Thermo Scientific) and analyzed using the neuronal profiling algorithm. The fold-change in MC1 intensity in neuritic processes relative to untreated wells was determined. The relative change with and without immunodepletion of rTg4510 extracts with respective antibodies was quantified. The degree of immunodepletion of total tau and p-tau were determined using sandwich ELISAs with HT7 as capture antibody and alkaline phosphatase-conjugated BT2, PHF13 or PHF6 as detection antibody as described in methods section on Tau ELISA assays.

### Tau transgenic mice

The rTg4510 mouse overexpressing the 0N4R P301L mutant form of human tau was used to evaluate the impact of anti-tau antibodies on age-dependent tau pathology [34]. rTg4510 mice have two transgenes: a tetracycline-controlled transcriptional activator (tTA) driven by the Ca^2+^/calmodulin-dependent protein kinase IIα promoter and a P301L tau transgene driven by a tetracycline operon-responsive element (TRE). Control mice or double negative mice (DN) contained neither the tTA nor the P301L tau transgene. The treatment studies utilized female rTg4510 mice, which exhibit robust age-dependent tau pathology over 3–6 months of age. PS19 mice, over-expressing the human T34 isoform of tau (1N4R) with the P301S mutation under control of the mouse prion promoter [35], were used in all the tau PFF-induced pathology studies [10]. PFF injection studies were done in male PS19 mice, since they exhibit more robust pathology than females [36]. Mice were housed with a 6:00 A.M. to 6:00 P.M. light/dark cycle and allowed free access to food and water.

### Ethics statement

All mice handling, surgeries and postoperative care procedures in this study, done at Bristol-Myers Squibb, were specifically approved by the Bristol-Myers Squibb Institutional Animal Care and Use Committee. All mice handling, surgeries and postoperative care procedures in this study, done at the University of Pennsylvania, were specifically approved by the University of Pennsylvania Institutional Animal Care and Use Committee.

### Intracerebral injections of K18PL PFFs in PS19 mice

A truncated form of human tau containing the four MT-binding repeats and the P301L mutation (K18PL), with or without a myc tag at the 5’-end, were produced from a pRK172 bacterial expression vector [10]. Purified K18PL peptide was fibrilized in the presence of heparin as described previously [19]. A robust Thioflavin T fluorescence signal is evident following fibrillization, with greater than 80% being recovered in the insoluble pellet fraction after ultracentrifugation compared to unfibrilized peptide. Aliquots of K18PL PFFs were stored frozen at -80°C, and sonicated briefly prior to injection into the brains of PS19 mice. All sterotaxic surgeries were performed in accordance with protocols approved by the Institutional Animal Care and Use Committee at Bristol-Myers Squibb (BMS) or the University of Pennsylvania. Male PS19 mice (2–6 months of age) were immobilized and deeply anesthetized with isoflurane gas coupled to the nose cone attached to a stereotaxic frame (David Kopf Instruments, CA) or by intraperitoneal (i.p) injection of ketamine hydrochloride (1 mg/10 g) and xylazine (0.1 mg/10 g). For studies done at BMS, stereotaxic injections were done with a 10 μL Hamilton syringe using a 30 gauge needle under aseptic conditions, into the hippocampus alone (bregma, −2.0 mm; lateral, + 1.6 mm; and depth, −1.7 mm from the surface of the brain). For studies done at the University of Pennsylvania, the protocol included both hippocampal and cortical injections with (bregma, −2.5 mm; lateral, + 2 mm; and depth, -1.8 mm, from the surface of the brain) for hippocampus, followed by an injection at -0.8 mm into cortex). Injections (2.5 μl) of either K18PL PFFs (2μg/μl) or PBS were made at all sites of injection. All injected mice were observed until they recovered from anesthetic, and an analgesic was administered once daily for 4 days post-surgery. Surgeries were highly successful with no mortality during the 4-week treatment. Mice were evaluated in behavioral assays in the final week before harvest.

### Antibody treatment

PHF13.6 and PHF6.10 hybridoma clones were grown in 20 liter cultures and antibody was purified from supernatants using HiTrap protein G column chromatography using an AKTA purification system (GE Healthcare, NJ). Antibody was eluted using 0.1M ethanolamine buffer (pH 11.5) and immediately neutralized with glycine buffer (1M Glycine HCl, pH 2.5). Samples were concentrated and buffer exchanged into PBS, followed by 0.2 μm filter sterilization. Endotoxin levels were <0.5 units/mg.

Female rTg4510 mice were dosed i.p. with 25 mg/Kg of PHF13.6, PHF6.10 or IgG2b (Bio X Cell, NH), once weekly for 12 weeks, from 3 to 6 months of age. Animals were euthanized 2 days after the final dose and plasma, CSF and brains were collected for analysis.

K18PL PFF-injected PS19 mice were dosed i.p. with 30 mg/Kg PHF13.6 or IgG2b starting on the day of surgery once weekly, for a total of 5 injections over 4 weeks duration. Mice were euthanized 2 days after the final dose and plasma and brains were collected for analysis.

### Behavioral assays

rTg4510 mice were evaluated in behavioral assays 11–12 weeks after treatment with anti-tau antibodies (~6 months of age). PS19 mice were tested in behavioral assays 4 weeks after K18PL PFF injections to compare the impact of anti-tau antibody vs. control antibody treatment. All tests were conducted blinded to treatment; distinct operators did the in-vivo dosing and the behavioral tests. Unblinding was done after completion of the behavioral analysis.

#### Elevated plus maze

Anxiety-like behavior was evaluated using an elevated plus maze assay. The maze consisted of two open and two enclosed arms elevated 65 cm above the ground. After acclimation to the dimly lit testing room for 60 min, mice were placed at the junction between the open and closed arms of the maze and allowed to explore for 5 min. After testing of each mouse, the maze was cleaned with 70% ethanol. The distance, entries and the time spent in each arm were recorded by a video camera mounted above the maze and analyzed using Topscan analysis system (Cleversys Inc., VA).

#### Spontaneous locomotor activity

Spontaneous activity was measured using an automated TruScan mouse activity monitor (25 cm long x 25 cm wide x 40 cm high; Coulbourn Instruments, Allentown, PA) equipped with a 16x16 photobeam system detecting horizontal and vertical movements. After acclimation to the testing room for 60 min, mice were individually placed into a test chamber and activity was recorded over 15 min. Data were collected at 2Hz and averaged over 1 minute bins. The apparatus was cleaned with 70% ethanol after evaluation of each mouse. Total movements (i.e., ambulation), rearing, and time spent in the center and periphery of the chamber were recorded automatically for subsequent analysis.

#### Novel object recognition

Plexiglass test chambers (40 cm long x 38 cm wide x 20 cm high) were used as the open field, with different-shaped objects (each 7.5 cm) as the test articles. Objects were placed 10 cm from the wall on diagonally opposing sides of the chamber. Mice were acclimated in the testing room for 60 min prior to training. Training comprised placing the mice in the Plexiglass test box with two identical objects and allowing the mice to explore the box and objects over 10 min. Activity was recorded using overhead cameras and analyzed with the TopScan Software (Cleversys Inc. Reston, VA). The number of approaches, time spent exploring each object and the total distance travelled in the chamber were recorded for subsequent data analysis. Exploration was scored positive if an animal’s nose was within 1cm of an object. Animals with a total object exploration time of <20 s during training were excluded in the final analysis. Arenas and objects were cleaned with 70% ethanol between the testing of each mouse. Recognition memory was tested an hour later, wherein each mouse was returned to the same chamber, presented with a familiar and a novel object and allowed to explore for 10 min. The number of approaches, time spent exploring each object and the total distance travelled in the chamber were recorded for subsequent data analysis. For each mouse, objects and their locations were randomly assigned. Total exploration over 10 min was calculated in PS19 mice, while in rTg4510 mice exploration over 5 min was analyzed due to reduced overall activity after that. Statistical analysis on percent time spent on novel object utilized an overall ANOVA followed by Bonferroni’s test or t-test versus control genotype or control treatment. Pairwise t-tests were used to evaluate differences between novel and familiar object within a group. All analyses were done in Prism (GraphPad, CA).

### Tissue harvest

Animals were euthanized by methods approved by the BMS Animal Care and Use Committee or those of the University of Pennsylvania Institutional Animal Care and Use Committee. CSF was collected from the cisterna magna immediately after blood collection by cardiac puncture [37]. Body temperature was maintained at normal levels during the collection procedure using heating pads. The cisterna magna was exposed by dissecting away the muscles on back of the neck, the dura was then punctured with a 30 gauge needle under a dissecting microscope, and CSF collected with a P20 pipettor. Following centrifugation at 1000 rpm for 10 min to remove any red blood cells, CSF was frozen on dry ice in low protein binding eppendorf tubes. For PS19 mouse studies, brains were removed and drop-fixed in freshly prepared 4% paraformaldehyde (BMS) or in 10% neutral buffered formalin (University of Pennsylvania). For rTg4510 studies, brains were hemisected, one hemisphere was fixed as above, while the second hemisphere was dissected to remove the hippocampus, anterior and posterior cortex and stored frozen until analysis.

### Antibody levels

Antibody levels in plasma and CSF were estimated using a one-sided ELISA assay that utilized a biotin-conjugated phosphorylated peptide immobilized on a Neutravidin plate, followed by alkaline phosphatase-conjugated anti-mouse detection antibody. For evaluating PHF13 levels, a biotinylated pS396 peptide (Biotin-HGAEIVYK-pS-PVVSGDT) corresponding to tau aa 388–403 was used as the capture peptide. For evaluating PHF6 levels, a biotinylated pT231 peptide (Biotin-TREPKKVAVVR-pT-PPKSPSSAKSR) corresponding to tau aa 220–240 was used as the capture peptide. Plasma samples were diluted at 1:10000 to 1:1,000,000, while CSF samples were diluted at 1:300 to 1:1000. Neutravidin plates (Thermo Scientific, MA) were coated with 50 μL of respective phospho-peptide at 100–300 ng/ml and incubated at RT for 1 hr, washed three times with PBS and blocked for 1 hr at RT with PBS containing 3% BSA. After removal of blocking agent, 50 μL of standards and samples were added, followed an hour later by alkaline phosphatase-conjugated goat-anti-mouse antibody. Following an hour of incubation at RT, plates were washed three times with PBS containing 0.05% tween. After a final wash, plates were tapped dry and developed using alkaline phosphatase substrate (T-2214, Life Technologies, CA) for 30 minutes. Luminescence counts were measured using an Envision plate reader (Perkin Elmer, MA). Raw counts were log-transformed, standard curves were fit using a 3^rd^ order polynomial and unknowns converted to antibody concentration.

### Brain extraction

Soluble and sarkosyl insoluble tau were isolated using a modification of a previously described procedure [37]. Briefly, hippocampi were homogenized in high-salt/sucrose buffer (10mM Tris-HCl, pH 7.4, 800mM NaCl, 10% sucrose (w/v), 1mM EGTA) supplemented with phosphatase inhibitor cocktail sets I and II (Calbiochem, CA), complete protease inhibitor cocktail EDTA-free (Roche, IN) and 1mM PMSF (Sigma, MO). Hippocampi were extracted in cold buffer (10X w/v) using a Potter-Elvehjem homogenizer. The extracts were then centrifuged at 20,000 x g for 20 minutes at 4°C. An aliquot of the soluble, supernatant fraction (300–400 μL) was stored frozen at -80°C while the slow-speed pellet was discarded. The remaining 200 μL of the supernatant was treated with Sarkosyl (N-lauroylsarcosine, Sigma L-7414), adjusted to 1% sarkosyl (v/v), and incubated for 60 min at 4°C with rocking. This extract was then centrifuged at 100,000×g for 1 h at 4°C and the resulting sarkosyl-insoluble pellet was rinsed using high salt/sucrose buffer and re-centrifuged. This pellet was then resuspended in 200 μL of 50mM Tris-HCl, pH 7.4 buffer containing 2.3% SDS, 1 mM EGTA, 1 mM EDTA, supplemented with phosphatase inhibitor cocktail sets I and II (Calbiochem, San Diego, CA), complete protease inhibitor cocktail EDTA-free (Roche, Indianapolis, IN) and 1mM PMSF (Sigma, St. Louis, MO). The pellet was sonicated in water bath for 30 minutes at RT and aliquots were stored frozen at -80°C. This comprised the sarkosyl-insoluble fraction.

### Brain and CSF Tau ELISA assays

Brain extracts were evaluated for total tau and pS202/pT205 tau levels, while CSF was evaluated for total tau and pT181 tau levels. The total tau and p-tau assays used the following mouse monoclonal antibodies (all from Thermo Scientific, MA); HT7 (aa 159–163, MN1000), BT2 (aa 194–198, MN1010), clone AT8 (pS202/pT205, MN1020), clone AT270 (pT181, MN1050). The determination of brain and CSF total tau levels utilized an ELISA assay with HT7 as capture antibody followed by detection with an alkaline phosphatase-conjugated BT2 antibody [38]. Brain pS202/pT205 was evaluated with an ELISA comprising an HT7 capture antibody followed by detection with alkaline phosphatase-conjugated AT8 antibody. CSF pT181 tau was assessed using an ELISA comprising an HT7 capture antibody followed by detection with alkaline phosphatase-conjugated AT270 antibody [38]. The capture antibody HT7 was used at a concentration of 1–2.5 μg/ml in all assays, while detection antibodies were directly conjugated with alkaline phosphatase (AP) (Novus Biologicals, CO).

Black 96-well plates (Corning-Costar 3925) were coated with 100 μL of respective capture antibodies in bicarbonate buffer (pH 9.4) (# 28382, Thermo Scientific, MA) overnight at 4°C. The following day, plates were washed with Dulbecco’s PBS (with Ca & Mg) four times, and then blocked with 3% BSA/DPBS for at least 3 hrs at room temperature (RT) or overnight at 4°C. For the total tau ELISA, recombinant Tau441 peptide standard (rPeptide, Bogart, GA) was utilized for determination of relative brain tau levels. For the HT7-AT8 ELISA, a custom standard was synthesized comprising the tau sequence from aa 153–211, with the S202 and T205 sites phosphorylated. For the HT7-pT181 tau ELISA, a standard was synthesized containing aa 155–207 of tau, with the T181 site phosphorylated. Standards were prepared in PBS supplemented with 0.1% BSA and 0.05% Tween. Brain and CSF samples were diluted so signal was in middle of the linear ranges for the respective assays. Standards and brain or CSF samples were added in duplicate at 50 μL/well, followed by AP-conjugated detection antibody (50 μL/well) in 3% BSA/PBS with 0.2% Tween 20. After overnight incubation at 4°C, plates were washed four times at RT with PBS containing 0.05% Tween. After the last wash, plates were developed with 100 μL of alkaline phosphatase substrate (T-2214, Life Technologies, CA) for 30 minutes. Luminescence counts were measured using an Envision plate reader (Perkin Elmer, MA). Raw counts were log-transformed, standard curves were fit using a 3^rd^ order polynomial and unknowns converted to respective tau or p-tau concentration. Brain tau and p-tau levels were dilution corrected and normalized to brain weight prior to further analysis. All graphs and statistics were done with Prism software (GraphPad, CA).

### Immunohistochemistry

For studies conducted at BMS, brain tissue was drop-fixed in 4% paraformaldehyde (PFA), processed through 15% and 30% sucrose, and cut in 40 μm horizontal sections using a sliding microtome. Sections were post-fixed in 3.7% formaldehyde in phosphate-buffered saline (PBS) for 10 minutes and washed using PBS. Endogenous peroxidase activity was removed by incubation in 3% hydrogen peroxide/10% methanol in PBS for 30 minutes. After washing in PBS, slides were blocked in 10% normal goat serum/0.3% Triton X-100 in PBS for one hour. Sections were stained with anti-pS202/pT205 tau antibody (AT8-biotin mouse IgG, 1:1,000, Thermo Scientific, Rockford, MD), conformational tau antibody MC1 (1:10,000 mouse IgG, kind gift from Dr. Peter Davies), an anti-Iba-1 antibody (mouse IgG, 1:60,000, Wako Chemicals USA, Richmond, VA), or an anti-glial fibrillary acidic protein (GFAP) antibody (polyclonal rabbit anti-GFAP, 1:20,000, Dako, Carpinteria, CA). Sections were stained with antibodies diluted in the blocking solution overnight at 4°C, washed with PBS and then incubated with either biotinylated anti-mouse or anti-rabbit IgG (Vector Labs, Burlingame, CA), except in the case of AT8-biotin, for 1 hour at room temperature. Slides were developed using a Vectastain ABC Elite Kit (Vector Labs, Burlingame, CA) for 1 hour followed by detection with diaminobenzidine reagent with nickel intensification. Nuclei were counterstained with hematoxylin-eosin stain.

For studies undertaken at the University of Pennsylvania, brains were drop-fixed in 10% neutral buffered formalin (NBF) and stored overnight at 4°C. To avoid overfixation, NBF was rinsed from the brain tissue the following day by exchanging 3x for 1hr in 50 mM Tris containing 150 mM NaCl. Each brain was cut into 2 mm coronal slices before paraffin embedding (Thermo-Shandon). Embedded tissue was cut into 6 μm coronal sections with a sliding microtome and dried overnight in an oven at 42°C. Sections were stained in a 1-in-5 series. Sections were deparaffinized in xylene and rehydrated in a descending series of ethanol. Endogenous peroxidase activity was quenched using methanol/hydrogen peroxide (150 ml methanol + 30 ml 30% hydrogen peroxide) for 30 min, followed by a 10 min rinse in running water and immersion in 0.1M Tris pH 7.6 buffer. Slides were blocked for 5 minutes in 2% fetal bovine serum in 0.1M Tris pH 7.6 buffer and incubated with AT8 (1:10,000) or MC1(1:7,000) antibodies diluted in blocking buffer overnight at 4°C. The rest of the staining procedure was performed the following day using a non-biotin polymer-HRP detection method with dioaminobenzidine (DAB) reagent and Myers hematoxylin counterstain (Biogenex).

### Image acquisition and analysis

For studies conducted at Bristol-Myers Squibb, brain sections mounted onto glass slides were imaged using an upright Nikon E800 or Leica DM6000 microscope equipped with a motorized XYZ stage (Prior Scientific, MA). The imaging system used a QiCam color, firewire-coupled camera to acquire images (QImaging, Canada). The microscope, stage and camera were controlled, via an Oasis PCI card using the Surveyor software (Objective imaging, Cambridge, UK). Autofocus parameters were setup, using four sites/brain section, followed by predictive autofocus during image acquisition. This enabled imaging of the entire slide of brain sections at optimal focus, typically 1–2 minutes/slide. In this study, ~ 2 slides from each animal were analyzed, each with 7–10 mounted horizontal brain sections for a total of 14–20 sections per animal. All slides from a study were imaged using identical illumination power and camera exposure. In addition, appropriate shading and color balance corrections were applied to every image. Following image acquisition, brain sections containing hippocampal and entorhinal cortex (EC) regions were identified for each animal and images exported for analysis. Hippocampal regions were selected from superior and inferior regions of horizontal brain sections (Bregma: -1.2 to -3.3 mm, Interaural: 4.6 to 2.5 mm; Horizontal brain atlas) [39], while medial and lateral EC brain regions were selected from inferior brain regions (Bregma: -2.2 to -3.8 mm, Interaural: 3.6 to 2.2 mm; Horizontal brain atlas) [39]. Images from individual brain sections were then analyzed using custom macros written in Imagepro Plus software (Media Cybernetics, MD). Identical threshold settings were used in all images to identify positively stained regions. Outlines were drawn around the ipsi and contralateral hippocampus and/or EC to restrict image analysis to these regions. For the hippocampus, overall area occupied by AT8 or MC1, and in the EC, total cell count and area occupied by AT8 or MC1 stain was quantified from at least 4–5 brain sections per animal.

For studies at the University of Pennsylvania, images from five sections in a 1-in-5 series from the anterior hippocampus of each mouse (approximately -2.0 to 2.5 mm from Bregma) were obtained at 4X magnification using an Olympus DP71 microscope. Analysis was performed using ImageJ software, where each image was converted to a 16-bit black-and-white image and subsequently thresholded using the MaxEntropy option. The area occupied by immunostaining after thresholding was determined using Image J software.

### Statistics

Statistical calculations were performed using GraphPad Prism 5.0 (GraphPad Software, San Diego, CA). Differences in endpoints between a treatment and control group were examined using unpaired two-tailed Student’s t-test. In experiments where there were greater than 2 treatment groups, significance versus the respective controls group was determined using ANOVA followed by Dunnett’s test. In experiments, with repeated comparisons such as in NOR, pairwise t-tests were performed on time spent on familiar versus novel objects.

## Results

### Immunodepletion with p-Tau antibodies reduces tau pathology in a primary neuronal model of tau aggregation

**Fig 1:**
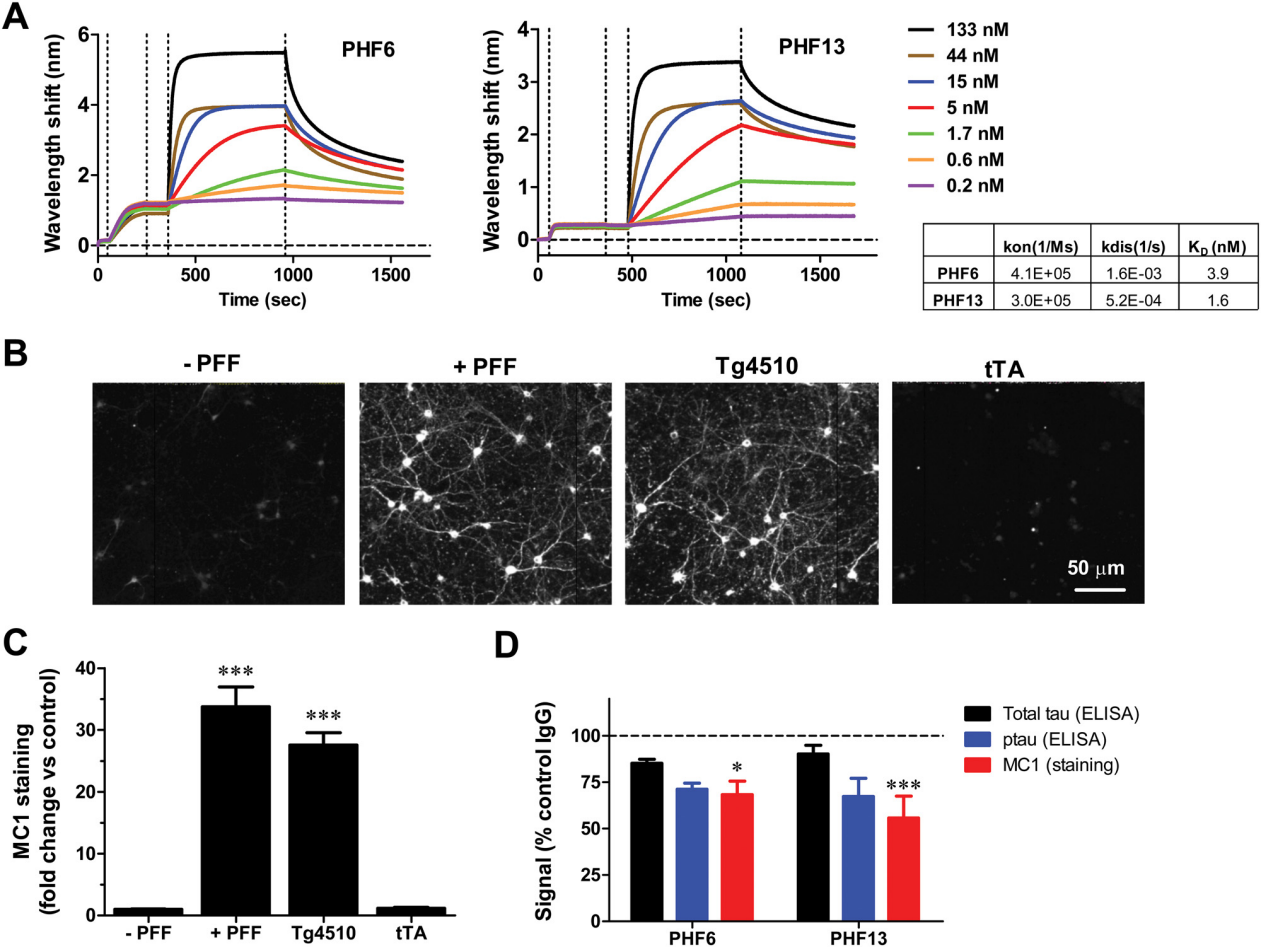
Immunodepletion with p-Tau antibodies reduces tau pathology in a primary neuronal model of tau aggregation. **A.** Kinetics of association and dissociation of PHF6 antibody and PHF13 antibody. The table summarizes the calculated association rates, dissociation rates, and K_D_ values for PHF6 and PHF13. The vertical dotted lines demarcate, from left to right—peptide loading, wash/equilibration, antibody association and antibody dissociation. **B.** Representative images from Triton-insoluble MC1 staining of primary neurons that received no PFF treatment (- PFF), treatment with 100 nM K18PL PFF (+ PFF), rTg4510 brain extract (Tg4510) or tTA brain extract (tTA). **C.** Change in Triton-insoluble MC1 staining intensity in primary neurons treated as in B, relative to untreated cultures (n = 6 wells/condition; ANOVA with Dunnett’s test vs. no treatment). **D.** Immunodepletion of rTg4510 extracts with PHF6 or PHF13 led to significant declines in Triton-insoluble MC1staining (Red bar) in neurons relative to control antibody (ANOVA with Dunnett’s test vs. Control IgG). Also displayed are total tau (black bar) and p-tau levels (blue bar) measured by ELISA after immunodepletion (*p<0.05, *** p<0.001, n = 3 per group).

Two distinct p-tau antibodies targeting pT231 (PHF6) and pS396 (PHF13) were selected for evaluation in a primary neuronal model of tau inclusion formation. These antibodies were derived from immunizations with AD brain-derived paired-helical filaments of tau and demonstrate robust labeling of pathological tau [40, 41]. The antibodies display comparable association rates onto immobilized peptides containing the appropriate p-tau epitope, but PHF6 exhibited ~3-fold faster dissociation rate constant than PHF13 (Fig 1A and 1B). The calculated affinity of PHF13 was 1.6 nM, whereas that of PHF6 was 3.9 nM.

The antibodies were assessed in a modified version of a previously described primary neuronal model of tau induction, wherein tau PFF treatment of P301S tau over-expressing neurons leads to an increase in detergent-insoluble tau aggregates that are positive for p202/p205 tau (recognized by AT8 antibody) and misfolded tau (recognized by MC1 antibody) [16]. Primary neurons from embryonic day 18 rat pups were infected at 4 days in vitro (DIV) with adeno-associated virus carrying a P301L full-length tau construct. A robust ~30-fold increase in MC1 staining was observed in both cell bodies and neurites after Triton X100 extraction and fixation of cells 7 days after treatment with 100 nM K18PL PFFs (Fig 1B and 1C; p<0.001). Treatment with soluble brain extract derived from 8-month old rTg4510 mice, which harbored substantial tau pathology, also led to a robust increase in MC1 positive, Triton-insoluble tau staining (Fig 1B and 1C; p<0.001). This effect was not observed when neuronal cultures were treated with brain extracts from control (tTA) mice (Fig 1B and 1C). These results reveal that misfolded tau species, either as synthetic fibrils or from aged rTg4510 brain extracts, can induce tau pathology in primary neurons over-expressing mutant tau.

The ability of PHF6 and PHF13 to prevent induction of tau pathology by rTg4510 brain extracts was subsequently evaluated in the primary neuron model. rTg4510 brain extracts were immunodepleted using 30 nM of control (26D6, anti-Aβ), PHF13 or PHF6 antibodies, and the non-bound fractions from brain extracts were then tested for their ability to induce pathology in the primary neuronal system. Immunodepletion of pS396 tau with PHF13 and pT231 tau with PHF6 led to a 30–45% decline in Triton-insoluble MC1 staining compared to control antibody (Fig 1D; PHF6 p<0.05, PHF13 p<0.001). A 30–40% reduction in respective p-tau levels (pT231 for PHF6 and pS396 for PHF13) and a small reduction in total tau levels were evident by ELISA (Fig 1D). Taken together, these results demonstrate that immunodepletion of tau containing pT231 or pS396 epitopes from brain extracts containing pathological tau species can reduce the induction of insoluble tau inclusions in primary neurons, and suggest that these antibodies may inhibit tau transmission in vivo.

### Age-dependent changes in tau pathology, brain and CSF tau /p-tau, in rTg4510 mice

**Fig 2:**
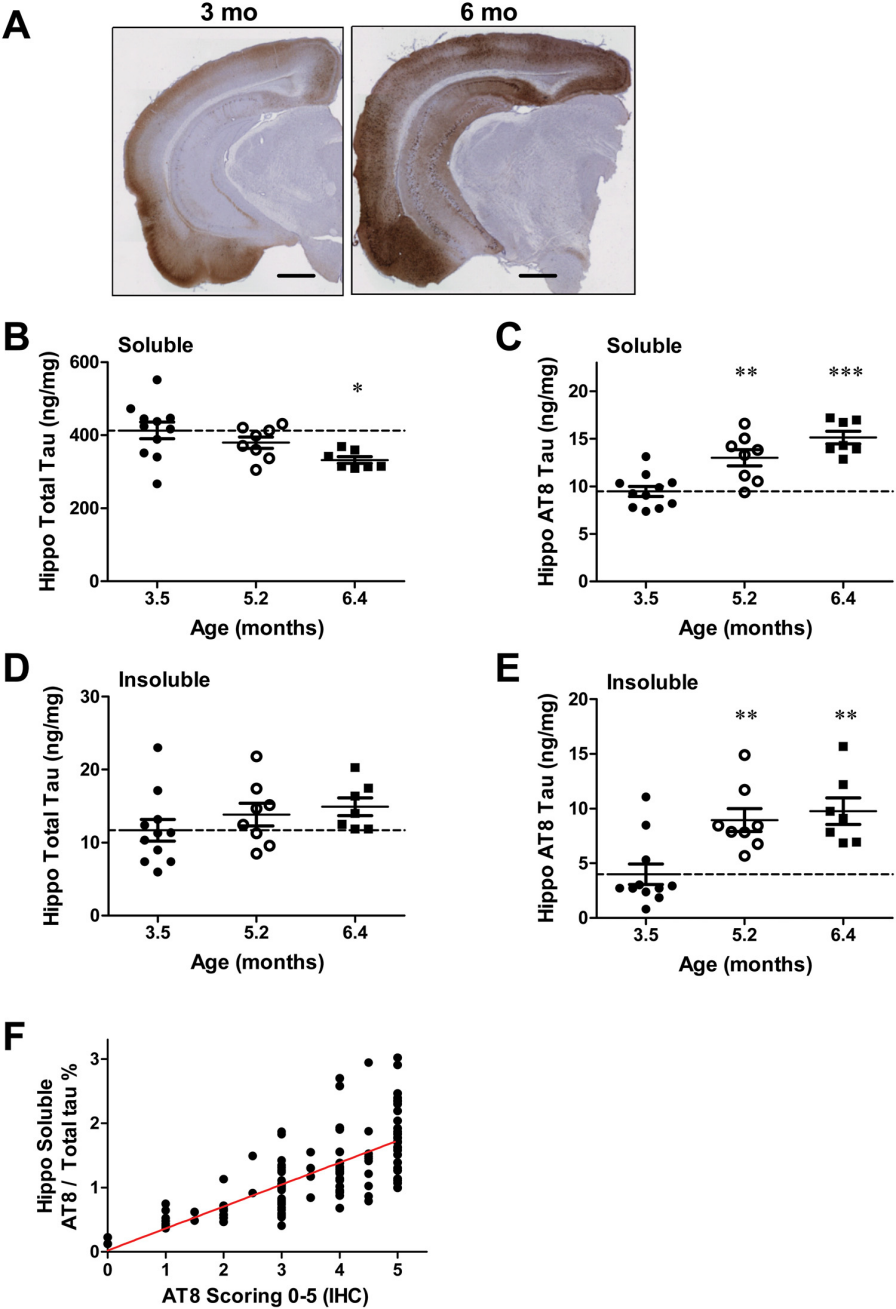
Age-dependent changes in tau pathology in rTg4510 mice. **A.** AT8 p-tau staining in 3- and 6-month old rTg4510 brain sections. Scale bar = 500 μm. **B-E.** Age-dependent change in **B.** hippocampal soluble total tau, **C.** hippocampal soluble AT8 p-tau, **D.** hippocampal insoluble total tau, and **E.** hippocampal insoluble AT8 p-tau. Individual comparisons were vs. 3.5-month old mice using ANOVA with a Dunnett’s post-hoc test (*p<0.05, ** p<0.01, *** p<0.001; n = 7–11 per group). **F.** Hippocampal soluble AT8 levels normalized to total tau levels were correlated with AT8 staining scores from immunohistochemistry (R^2^ = 0.51, p<0.001).

As the PHF6 and PHF13 antibodies reduced tau inclusion formation in tau-expressing primary neurons that were seeded with rTg4510 brain extracts, it was reasoned that these antibodies may be capable of attenuating the development of pathological tau species in the rTg4510 mice. In anticipation of these studies, we evaluated AT8 p-tau staining and the levels of tau and p-tau in soluble and insoluble brain extracts, as well as in CSF, as a function of age in rTg4510 mice. A substantial age-dependent increase in AT8 staining was observed from 3 to 6.5 months (Fig 2A). In 3-month-old mice, AT8 staining was apparent in cortex, with minimal staining in the hippocampus that was localized to cell bodies in the CA3 region. By 6 months of age, intense AT8 staining was evident in the cortex, along with extensive somatodendritic labeling in the CA3 and CA1 regions of the hippocampus (Fig 2A).

Total tau and p-tau levels in the hippocampus from rTg4510 mice were quantified as a function of age using quantitative ELISA’s. A small but significant decline (~20%) in brain soluble total tau was evident at 6.4 months of age when compared to 3.5 months of age as has previously been described [34, 37] (Fig 2B; p<0.05). A specific and sensitive ELISA was developed for evaluation of brain AT8 p-tau levels (S1 Fig). A significant age-dependent increase in soluble AT8 tau levels was apparent at 5.2 and 6.4 months of age when compared to 3.5 months of age (37%, p<0.01 and 59%, p<0.001, increases, respectively; Fig 2C). An increase in both total tau and AT8 p-tau was observed with age in detergent-insoluble brain extracts (Fig 2D and 2E). Whereas insoluble total tau showed a 10–20% increase from 3–6 months of age (Fig 2D), brain insoluble AT8 p-tau levels increased two-fold at 5–6 months of age when compared to 3-month-old mice (Fig 2E; p<0.01). We also observed a significant correlation between relative AT8 staining by IHC and soluble AT8 levels normalized to total tau in individual rTg4510 mice with age (Fig 2F; R^2^ = 0.51, p<0.001). These results demonstrate a robust age-dependent increase in brain AT8 p-tau levels, concurrent with the increase in AT8 pathology.

**Fig 3:**
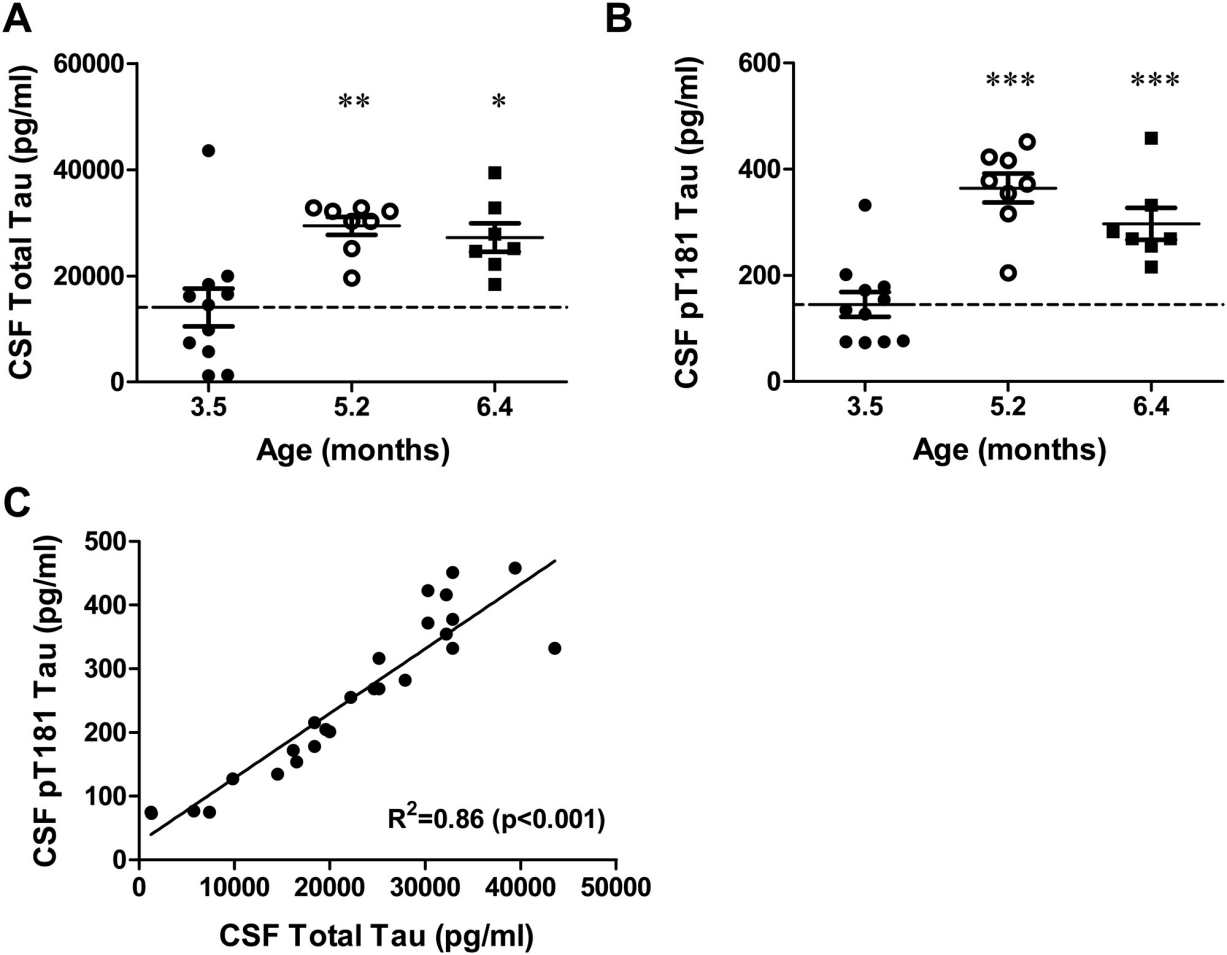
Age-dependent changes in CSF tau and pT181 tau in rTg4510 mice. Age-dependent increases in **A.** total tau and **B.** pT181 tau are observed in CSF from rTg4510 mice. Individual comparisons vs. 3.5 month old mice using ANOVA with a Dunnett’s post-hoc test (*p<0.05, ** p<0.01, *** p<0.001; n = 7–11 per group). **C.** CSF total tau was highly correlated with CSF pT181 tau (R^2^ = 0.86, p<0.001).

CSF tau levels were also examined in rTg4510 mice, and increases in CSF total tau and p-tau were observed as the mice aged (Fig 3A). A ~2-fold increase was evident at 5.2 vs. 3.5 months of age, with no further increase at 6.4 months of age, consistent with previous results [37] (5.2 mo p<0.01, 6.4 mo p<0.05). In AD subjects, increases in CSF pT181 and total tau levels have been demonstrated to be an early biomarker of disease onset and progression [42]. Therefore, we developed a sensitive CSF pT181 tau ELISA to measure p-tau from aging rTg4510 mice and to evaluate the impact of p-tau antibody treatment (S2 Fig [38]. A 2-fold increase in CSF pT181 tau levels was observed in older rTg4510 mice (Fig 3B; 5.2 mo p<0.001, 6.4 mo p<0.001), with CSF pT181 tau levels highly correlated with CSF total tau levels (Fig 3C; R^2^ = 0.86, p<0.001). CSF total tau and pT181 tau levels also showed correlation with brain soluble AT8, brain insoluble AT8 tau and brain insoluble total tau (Table 1). In addition, brain soluble AT8 tau levels were correlated with brain soluble and insoluble total tau, and insoluble AT8 tau levels (Table 1). These results demonstrate an age-dependent increase in soluble and insoluble AT8 tau in the rTg4510 mouse brain, which is associated with increases in CSF total tau and pT181 tau levels.

**Table 1:** Correlations between brain and CSF tau and p-tau levels.

Analyte 1	Analyte 2	R^2^	p value
Brain Sol. AT8	Brain Sol. Total Tau	0.21	0.023
	Brain Insol. Total Tau	0.27	0.006
	Brain Insol. AT8 Tau	0.62	< 0.0001
CSF Total Tau	Brain Sol. AT8	0.40	0.001
	Brain Sol. Total Tau	0.05	0.282
	Brain Insol. Total Tau	0.27	0.006
	Brain Insol. AT8 Tau	0.57	< 0.0001
	CSF pT181 Tau	0.86	< 0.0001
CSF pT181 Tau	Brain Sol. AT8	0.43	0.0003
	Brain Sol. Total Tau	0.13	0.081
	Brain Insol. Total Tau	0.15	0.053
	Brain Insol. AT8 Tau	0.56	< 0.0001

### p-Tau antibodies reduce brain soluble AT8 and CSF p-tau, and result in functional improvement in rTg4510 mice

The PHF6 and PHF13 antibodies that caused a reduction in tau pathology in primary neurons (Fig 1E) were evaluated in rTg4510 mice to determine whether they might alter brain or CSF tau or p-tau levels. rTg4510 mice were treated with PHF13, PHF6 or IgG2b isotype control antibodies at 25 mg/Kg i.p. once weekly, starting at 3 months of age through 6 months of age. Analysis of plasma PHF13 and PHF6 levels after 12 weeks of dosing revealed 100–500 μg/ml (0.7–3.5 μM), while CSF antibody levels were in the range of 150–500 ng/ml (1–3.5 nM) (summarized in Table 2). The CSF/plasma ratio was ~0.2%, consistent with CSF exposure for antibodies in human studies [43].

**Table 2:** Antibody exposure in plasma and CSF.

Genotype	Treatment duration & age range (mo)	Antibody	Dose (mpk)	Plasma (μM)		CSF (nM)		CSF/plasma ratio %
				Mean	%CV	Mean	%CV	
**rTg4510**	3 (3–6)	PHF6	25	2.2	27	1.7	39	0.08
		PHF13	25	1.3	40	2	33	0.16
**PS19**	1 (2.5–3.5)	PHF13	30	1.6	49	1.6	21	0.1

**Fig 4:**
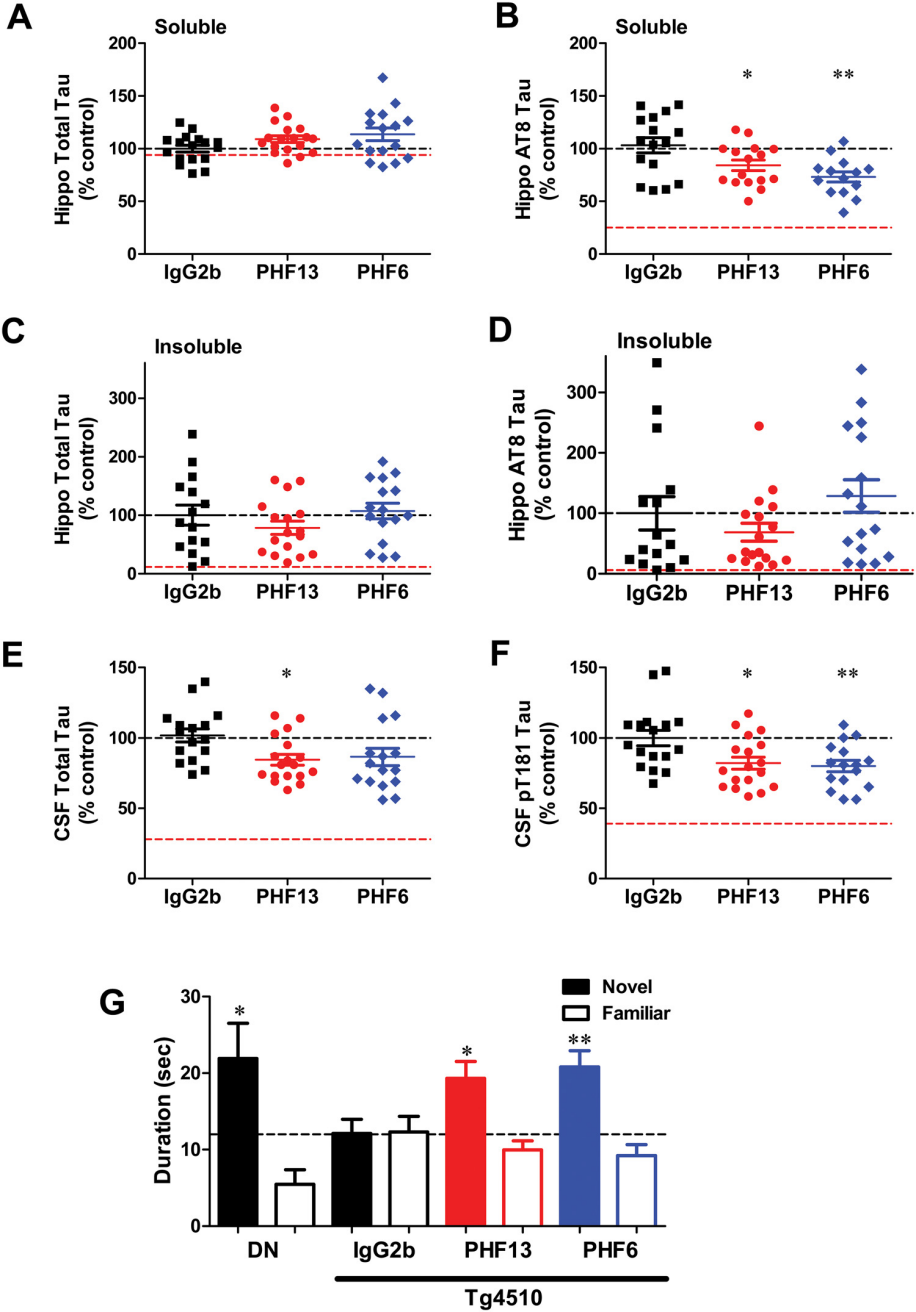
p-Tau antibodies reduce brain p202/p205 tau and CSF p181 tau with improved NOR performance. Effect of PHF-13 and PHF-6 (25 mg/Kg i.p.) on **A.** hippocampal soluble total tau; **B.** hippocampal soluble AT8 (p202/p205) tau; **C.** hippocampal insoluble total tau; **D.** hippocampal insoluble AT8 tau; **E.** CSF total tau; and **F.** CSF pT181 tau. Data were analyzed by ANOVA with post-hoc comparisons to IgG2b-treated mice using Dunnett’s test (*p<0.05, ** p<0.01; n = 14-18/ group). Red dashed lines indicate tau levels at the start of treatment (3-months of age). **G.** Effect of p-tau antibodies on NOR performance. The total time spent on novel (filled bar) and familiar objects (open bar) are plotted for each treatment group. DN represents double-negative (-tTA and—tau) mice. Statistical comparisons between the time spent on novel and familiar objects for each animal in a group was performed using a pairwise T-test. (DN, p<0.05; IgG2b p = 0.8; PHF13, p<0.05; PHF6, p<0.01; n = 6-8/group).

Although treatment with PHF13 or PHF6 did not lead to significant changes in brain soluble total tau (Fig 4A), a significant decline in brain soluble AT8 tau was observed after both antibody treatments (Fig 4B; PHF13 p<0.05, PHF6 p<0.01). No significant changes were evident in either insoluble total tau or AT8 p-tau after PHF13 or PHF6 treatment (Fig 4C and 4D). Consistent with the lack of change in insoluble AT8 p-tau, no significant change was observed in AT8 immunostaining in the brains of the rTg4510 mice (S3 Fig). We also evaluated a panel of genes comprising microglial and immune related markers by RT-PCR. An age and gender-dependent increase in different markers was evident in rTg4510 mice, but no significant changes in these genes were observed upon PHF6 or PHF13 treatment (S1 Table).

Interestingly, a significant reduction in CSF total tau was produced by PHF13 treatment (p<0.05), with a trend for a decline in the PHF6-treated mice (Fig 4E). Significant reductions in CSF pT181 tau levels were also observed in both the PHF13 and PHF6 treatment groups (Fig 4F; PHF13 p<0.05, PHF6 p<0.01). The reductions in brain soluble AT8 p-tau, and CSF total tau and p-tau in the mice receiving PHF6 and PHF13 represented a 20–40% decrease in the age-dependent elevation of these markers observed in control mice from 3 to 6 months of age (levels at start of treatment indicated by red dashed lines in Fig 4). The decline in brain soluble AT8 p-tau levels and CSF pT181 tau levels were not due to direct interference by the PHF13 or PHF6 antibodies in the respective p-tau assays, as these antibodies did not affect the ELISA readings at greater than 10x CSF exposure levels (S4, S5 and S6 Figs).

It is known that rTg4510 mice develop cognitive deficits as a function of age, and there is evidence that oligomeric species of tau may affect learning and memory in these mice [34]. Although there were not significant differences in insoluble tau levels in the brains of the PHF6- and PHF13-treated rTg4510 mice, the reduction of soluble AT8 p-tau in the brain, and total tau and p-tau in the CSF, suggested the possibility that soluble pathogenic tau species could be lowered by antibody treatment and show functional benefits. We therefore evaluated NOR in rTg4510 mice treated with IgG2b, PHF6 and PHF13, and compared them with the DN mice. rTg4510 mice treated with IgG2b showed no difference in time spent on novel and familiar objects, demonstrating a deficit in learning when compared to control DN mice, which spend a significantly greater time exploring the novel object (p<0.05;Fig 4G). Treatment with PHF13 and PHF6 led to an improvement in the total time spent on the novel object compared to the familiar object (Fig 4G; PHF13 p<0.05, PHF6 p<0.01), with performance approaching that of the DN mice. We also tested animals in a locomotor activity assay and the elevated plus maze. While rTg4510 mice exhibited greater locomotor activity and spent more time on the open arms of the elevated plus maze compared to DN mice, there was no significant impact of either antibody on these endpoints (data not shown). Taken together, these results demonstrate that treatment of rTg4510 mice with either PHF6 or PHF13 antibody results in a significant reduction in soluble brain AT8 and CSF tau, with functional improvement in NOR memory.

### p-Tau antibody treatment reduces tau pathology and leads to functional improvement in PS19 mice injected with tau PFFs

A growing body of evidence reveals that the development and distribution of tau pathology can be hastened substantially in tau transgenic mice through the injection of brain homogenates prepared from tau transgenic mice with existing NFT-like inclusions [9]. These studies have bolstered the hypothesis that tau pathology can be transmitted from one neuron to another, possibly through existing neural networks. One month after intra-hippocampal injection of K18PL PFFs into young PS19 mice [35], significant increases in p-tau (AT8 antibody) and misfolded tau (MC1 antibody) immunostaining were evident in both the ipsilateral and contralateral hippocampus, and bilaterally in the EC and locus coeruleus [10], at an age when there is normally no p-tau pathology. These data suggest that tau pathology spreads into regions distant from the PFF injection site. We therefore utilized this model of pathological tau induction and transmission to test the consequences of PHF13 immunotherapy on tau pathology and novel-object recognition memory.

**Fig 5:**
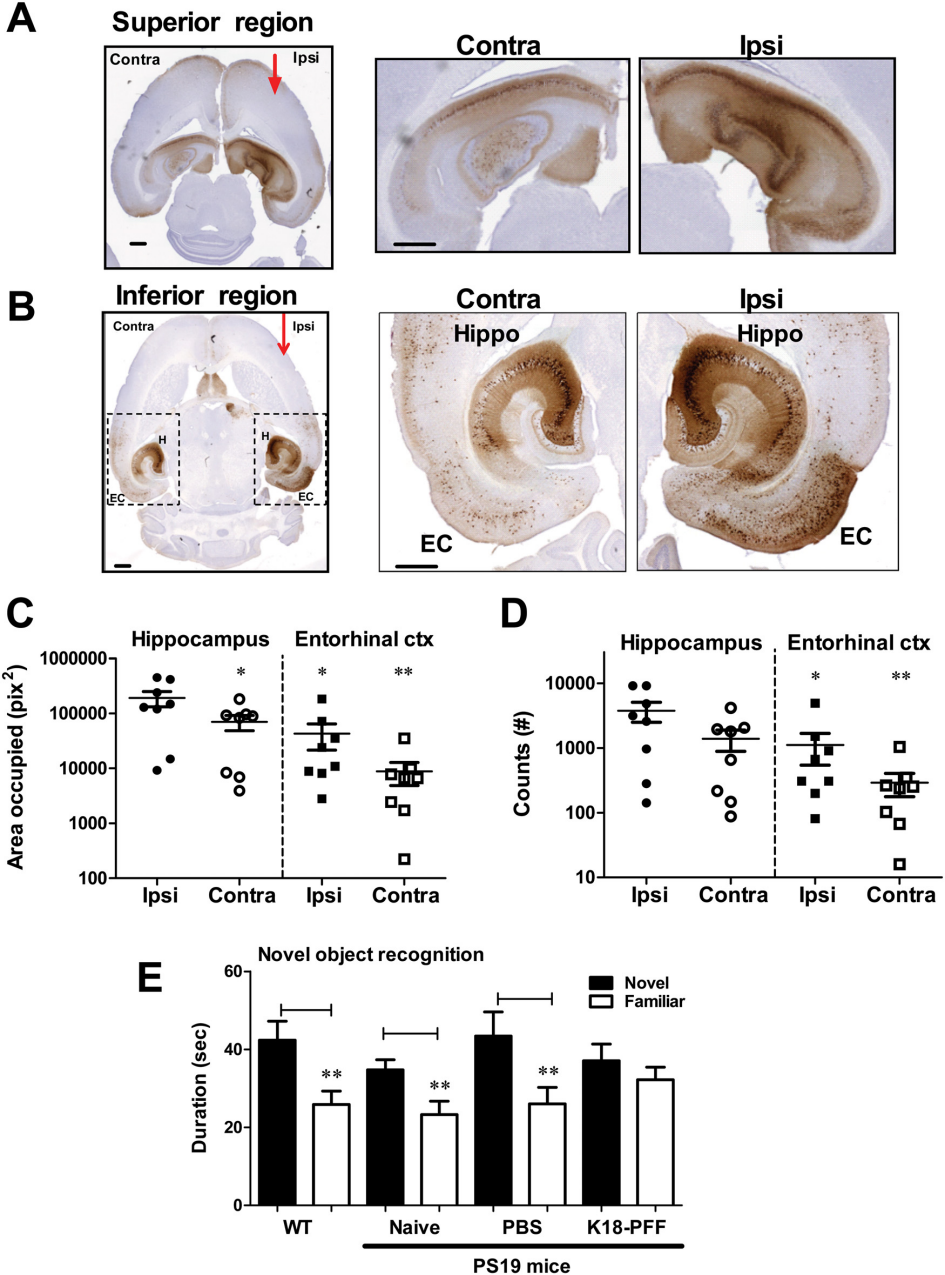
Hippocampal injection of K18PL PFFs leads to robust tau pathology in young PS19 mice. K18PL PFFs were injected into the hippocampus and mice evaluated 4 weeks later. **A.** Robust hippocampal AT8 staining on the ipsilateral (Ipsi) side of the PFF injection and on the opposite contralateral (Contra) side at a superior horizontal plane. Right panels show enlarged views of ipsilateral and contralateral hippocampi. Scale bar is 500 μm. **B.** Distinct AT8 staining in the hippocampus (H) and in the entorhinal cortex (EC) in regions inferior to the site of PFF injections. Right panels show enlarged views of the ipsilateral and contralateral hippocampi and EC. Scale bar is 500 μm. **C.** Area occupied by AT8 p-tau immunostaining in the ipsilateral and contralateral sides of the hippocampus and EC. **D.** Cell body counts positive for AT8 p-tau in the ipsilateral and contralateral sides of the hippocampus and EC. Statistical analyses in C. and D, compared all groups to ipsilateral hippocampus, the site of PFF injection, by ANOVA followed by Dunnett’s test (* p<0.05, ** p<0.01). **E.** Effect of genotype and PFF injections on novel object recognition performance. The total time spent on novel (filled bar) and familiar objects (open bar) are plotted for each treatment group. The different groups included WT, naive (un-injected), and PBS- or K18PL PFF-injected PS19 mice. Statistical comparisons between the time spent on novel and familiar objects for each animal in a group was performed using a pairwise T-test. (** p<0.01; n = 8-14/group).

To further characterize this model prior to utilization in immunotherapy studies, PS19 mice were injected with 5 μg of K18PL PFFs into the hippocampus at 2.5 months of age and tissues were harvested 4 weeks later. A robust spread of AT8 pathology through different brain regions was observed relative to the site of injection of PFFs (S7 Fig), with significant AT8 tau pathology in the ipsilateral and contralateral hippocampus in the superior horizontal plane (Bregma -1.8 mm, Interaural 4.0 mm; horizontal brain atlas [39]) (Fig 5A). No AT8 or MC1 pathology was apparent in PBS injected animals (S7 Fig). In a more inferior horizontal plane (Bregma -3.3 mm, Interaural 2.5 mm), intense AT8 staining was evident in the ipsilateral and contralateral hippocampus and in bilateral entorhinal cortex (EC) (Fig 5B). Robust cell body and some neuritic staining were evident in the CA3 and in the CA1 regions of the ipsilateral hippocampus, with a corresponding but lower intensity in the contralateral CA3 and CA1 regions. AT8 staining was apparent in layer 2/3 neurons in the medial and lateral EC bilaterally (Fig 5B). In addition to the EC, distinct AT8 staining was apparent in the lateral and medial septal nuclei along the midline (Fig 5B). We quantified tau pathology by evaluating both area occupied by AT8 staining and the number of cells positive for AT8 staining in the hippocampus and EC (S7 Fig). The total area occupied by AT8 was ~5-fold greater in the ipsilateral hippocampus compared to the contralateral hippocampus (Fig 5C; p<0.05). Likewise, the area occupied by AT8 immunoreactivity was 5–10 fold higher in the ipsilateral hippocampus compared to the ipsilateral EC (Fig 5C; p<0.05). Both the number of cells positive for AT8 and the area occupied by AT8 staining was significantly greater in the ipsilateral EC compared to the contralateral EC (Fig 5C and 5D; p<0.01). These results reveal a robust spread of K18PL PFF-driven tau pathology from the hippocampus to the EC, the contralateral hippocampus and other distant brain regions.

Analysis of CSF from K18PL PFF-injected PS19 mice revealed a small but statistically significant increase in tau relative to PBS-injected mice (S8 Fig). The lack of a robust increase in CSF tau in the K18PL PFF-injected mice, as was seen in the aged Tg4510 mice (Fig 3), likely relates to the greater extent of overall cerebral tau pathology in the older Tg4510 mice compared to the more regionally restricted tau pathology observed in the young PS19 mice that were inoculated with K18PL PFFs.

The impact on cognitive performance caused by the rapid onset of hippocampal and EC tau pathology following K18PL PFF injections into young PS19 mice was evaluated using the NOR test. Age-matched WT littermates from the PS19 cohort showed significantly greater time spent exploring the novel object vs. the familiar object (Fig 5E; p<0.01). Likewise, naïve and PBS-injected PS19 mice showed a preference for novel object exploration at this age (Fig 5E; p<0.01). In contrast, K18PL PFF-injected PS19 mice were impaired and showed no difference in the time spent on the novel vs. the familiar object (Fig 5E). These results demonstrate that the induction and spread of tau pathology after K18PL-PFF injection into the cortex and hippocampus of young PS19 mice resulted in a functional deficit in NOR.

**Fig 6:**
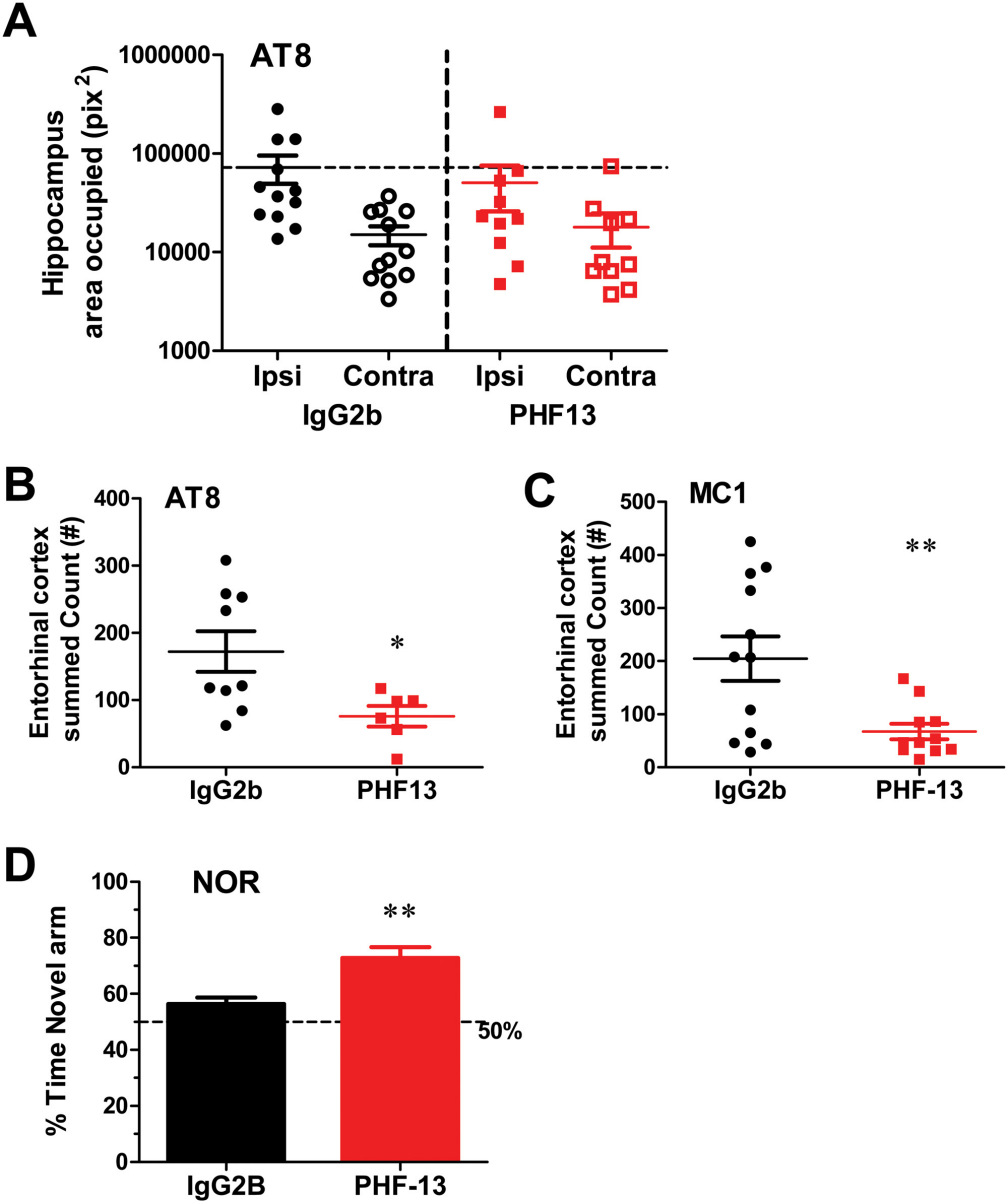
PHF13 reduces EC tau pathology and rescues NOR performance in PS19 mice injected with K18PL PFFs. K18PL PFFs were injected into the hippocampus and mice evaluated following treatment with PHF13 or IgG2b for 4 weeks. **A.** Hippocampal area occupied by AT8 immunostaining on the ipsilateral (Ipsi) and contralateral (Contra) sides following treatment with 30 mg/Kg i.p. IgG2b or PHF13. **B.** AT8-positive cell counts within the EC of IgG2b- and PHF13-treated mice. **C.** MC1-positive cell counts within the EC of IgG2b- and PHF13-treated mice. **D.** Percent time spent on the novel object in a NOR assay. Statistical analyses were performed using a t-test comparison between IgG2b and PHF13 treatment groups (* p<0.05, ** p<0.01; n = 10-12/group).

The extent and distribution of tau pathology that was observed in the young PS19 mice following K18PL PFF injection, coupled with the resulting deficit in NOR, confirmed that this model could be of value in evaluating the efficacy of p-tau antibodies in abrogating the spread of pathological tau. Young 2–3 month old PS19 mice were thus treated with PHF13 or IgG2b at 30 mg/Kg i.p. for 4 weeks starting on the day of K18PL PFF injections (for a total of 5 doses) to evaluate the impact of treatment on tau pathology and performance in the NOR task. It should be noted that the PHF13 antibody does not interact with the K18PL PFFs that are used to induce brain pathology in the PS19 mice, as this recombinant protein does not contain the pS396 epitope. Thus, PHF13 treatment would not be expected to inhibit the initial seeding of tau pathology in neurons that could internalize the injected K18PL PFFs. Plasma PHF13 levels measured at the time of sacrifice were in the range of 1–2 μM, while CSF PHF13 levels were 1–2 nM (Table 2). Robust increases in hippocampal AT8 tau pathology were evident in both the IgG2b- and PHF13-treated mice, with ipsilateral pathology being 3–5 fold higher than that in the contralateral side (Fig 6A). No significant differences were observed between the PHF13 and IgG2b treatment groups in the area occupied in the ipsilateral or contralateral hippocampal area occupied by AT8 (Fig 6A) immunostaining. Total AT8- and MC1-positive cell counts were derived from both ipsi and contralateral EC, since these represent sites of spread of tau pathology following injection of the PFFs. A significant decline in AT8 (p<0.05) and MC1 positive counts (p<0.01) was evident within the EC after PHF13 treatment relative to the IgG2b group (Fig 6B and 6C). These results demonstrate a reduction in the spread of tau pathology from the hippocampus to the EC following treatment with PHF13. The impact of PHF13 treatment on NOR was also assessed; PHF13-treated mice spent a significantly greater percentage of time on the novel object compared to the mice receiving IgG2b treatment (Fig 6D; p<0.01).

**Fig 7:**
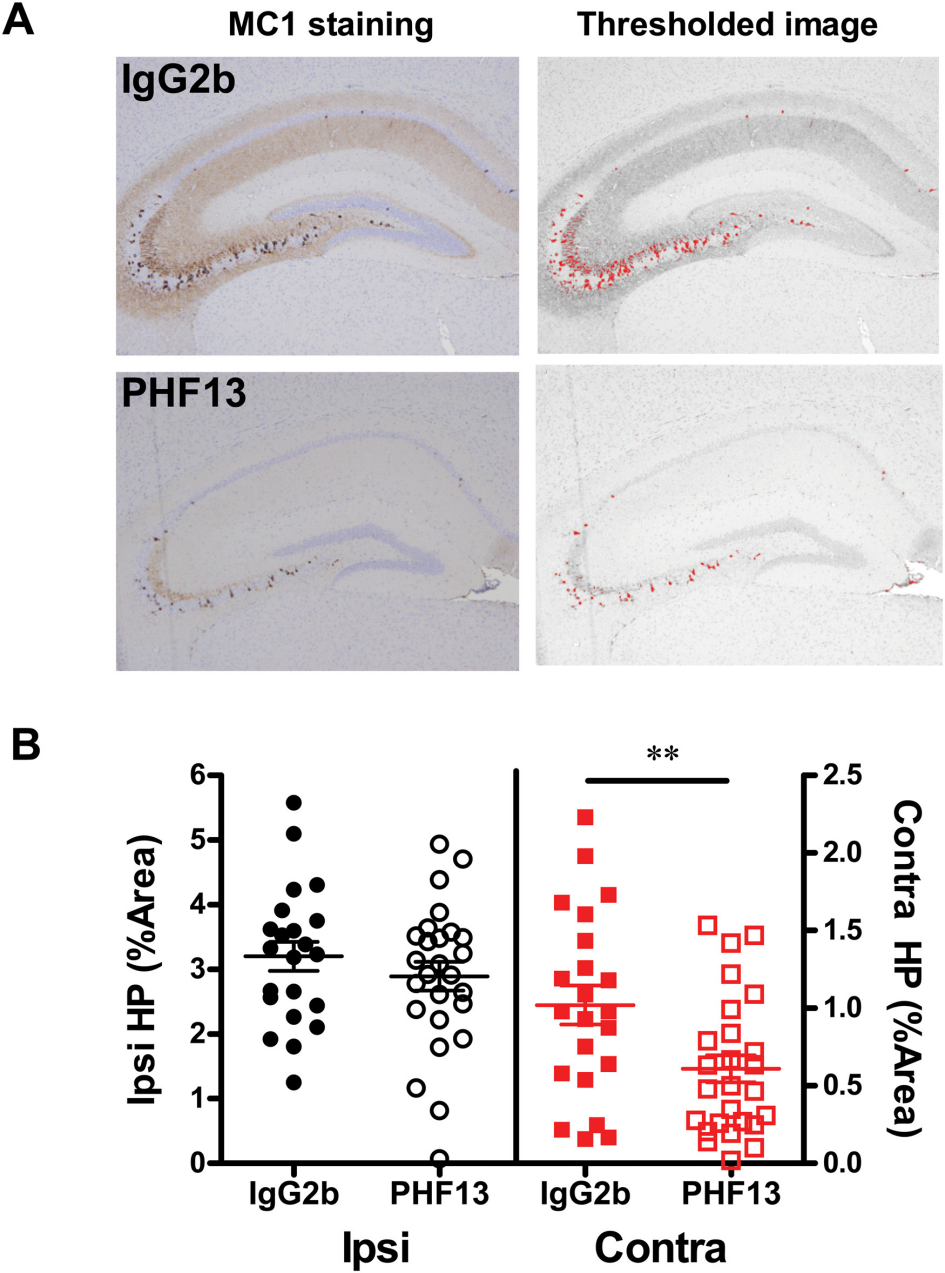
PHF13 reduces contralateral hippocampal tau pathology in PS19 mice injected with K18PL PFFs. The extent of hippocampal MC1-positive tau pathology was evaluated in PS19 mice that received K18PL PFF injections into both the hippocampus and overlying cortex and treatment with either IgG2b or PHF13 (30 mpk i.p. for 4 weeks). **A.** Contralateral hippocampal images from coronal sections stained with MC1 from mice treated with either IgG2b (top panels) or PHF13 (bottom panels). Right panels show images after non-biased thresholding to identify MC1-positive pathology for quantification. **B.** The percent of hippocampal area occupied by MC1 staining from IgG2b- and PHF13-treated mice. The left Y-axis and panels (black symbols) show ipsilateral (Ipsi) hippocampal (HP) % area, whereas the right Y-axis and panels (red symbols) show contralateral (Contra) hippocampal % area. Statistical analyses were based on t-test comparisons between IgG2b and PHF13 treatment groups (** p<0.01).

To further evaluate the effect of passive immunization with PHF13 in K18PL PFF-injected PS19 mice, additional studies were undertaken by a different research team (University of Pennsylvania). Two large experiments with similar design were conducted in which PS19 mice ranging from 2–6 months of age were dosed weekly starting on the day of K18PL PFF injection with 30 mg/kg of PHF13 or control IgG2b once-weekly for 4 weeks. As the contralateral hippocampus represents an area of relatively distant tau pathological spread across hemispheres, this region was the focus of these additional analyses. Misfolded pathologic tau was assessed by MC1 immunohistochemical analysis of a series of coronal sections that spanned the entire ipsilateral and contralateral hippocampus, with a determination of the average area occupied per section after automatic thresholding (Fig 7A). When the results of the two large, but similarly designed studies, were combined (resulting in 22–26 mice/treatment), a significant reduction of MC1-positive tau pathology was observed in the contralateral hippocampus of the PHF13-treated PS19 mice relative to the IgG2b-treated control group (p<0.01), despite the relatively large variation in the extent of hippocampal tau pathology (Fig 7B). There was no difference in MC1 immunostaining between the two groups in the ipsilateral hippocampus. The lack of an antibody-mediated decrease in AT8 (Fig 6A) or MC1 (Fig 7B) immunostaining in the ipsilateral hippocampus is likely due to the rapid and robust induction of pathology in this region that results from the internalization of K18PL PFFs, which as noted are not recognized by the PHF13 antibody. When the results of the studies with K18PL PFF-injected young PS19 from both laboratories are considered in total, there is evidence that PHF13 treatment can reduce tau pathology in the EC and the contralateral hippocampus in a model in which there is rapid spreading of tau pathology from the initial sites of PFF injection, with a resulting improvement of performance in the NOR test.

## Discussion

Antibodies targeting the pT231 and pS396 epitopes of tau, with affinities of 1–4 nM, were evaluated in a primary neuron culture model of tau pathology, as well as in two distinct tau transgenic mouse models that develop prominent tau inclusions with aging that are very reminiscent of those seen in AD and related tauopathies. Immunodepletion of p-tau species from brain extracts derived from aged rTg4510 mice with PHF13 or PHF6 reduced the ability of these preparations to induce tau pathology in primary neurons overexpressing mutant P301L tau. Moreover, both of these p-tau antibodies reduced p-tau species in vivo, with a concomitant improvement in cognitive performance. In this regard, both PHF13 and PHF6 were evaluated in rTg4510 mice, which over-express inducible human P301L tau and show an age-dependent accumulation of tau pathology, as well as an increase in CSF tau and p-tau. We observed a significant correlation of brain soluble and insoluble AT8 p-tau with CSF total tau and pT181 p-tau in the rTg4510 mice. These age-dependent increases of CSF markers occur in concert with a decline in brain soluble tau levels and increased insoluble AT8 tau. Thus, changes in CSF tau and p-tau may reflect the onset and spread of brain tau pathology and may provide a surrogate read of overall brain tau pathology.

Notably, treatment of rTg4510 mice with either PHF6 or PHF13 resulted in an attenuation of soluble AT8-immunoreactive p-tau in the brain, as well as decreased CSF total tau and pT181 tau. Treatment with an N-terminal specific antibody was shown to specifically reduce N-terminal- containing tau fragments in the brain interstitial fluid (ISF) and CSF [44]. However, the impact of antibody treatment on total tau or p-tau levels was not evaluated. This is the first study to demonstrate significant reduction in CSF total tau and p-tau levels following tau antibody treatment. However, there were minimal effects of these treatments on insoluble tau. This raises the possibility that the increase in insoluble tau in the rTg4510 mice may be driven largely by cell-autonomous tau overexpression, with antibodies having a nominal impact on the intracellular insoluble pool. Thus, the temporal progression of pathology from hippocampal to cortical regions that is observed with age in the rTg4510 mice may not be due to transmission of tau pathology, but rather to differences in the level of tau expression or in the intracellular environment which affects the rate of tau inclusion formation in distinct neuronal populations. Nonetheless, improvements in NOR performance were observed after treatment with the p-tau antibodies, which is consistent with findings in rTg4510 mice where suppression of tau expression by doxycycline treatment led to significant lowering of soluble tau and behavioral improvements in the absence of a significant change in tau NFT pathology [34]. Recent studies demonstrate that mutant tau overexpression specifically in the EC led to synaptic deficits within the entorhinal-hippocampal network prior to tau aggregation and overt neuronal loss [45]. This, along with studies in drosophila tau-overexpression models [46], suggests that soluble tau forms may be detrimental and insoluble tau aggregates may act to sequester toxic tau species.

**Fig 8:**
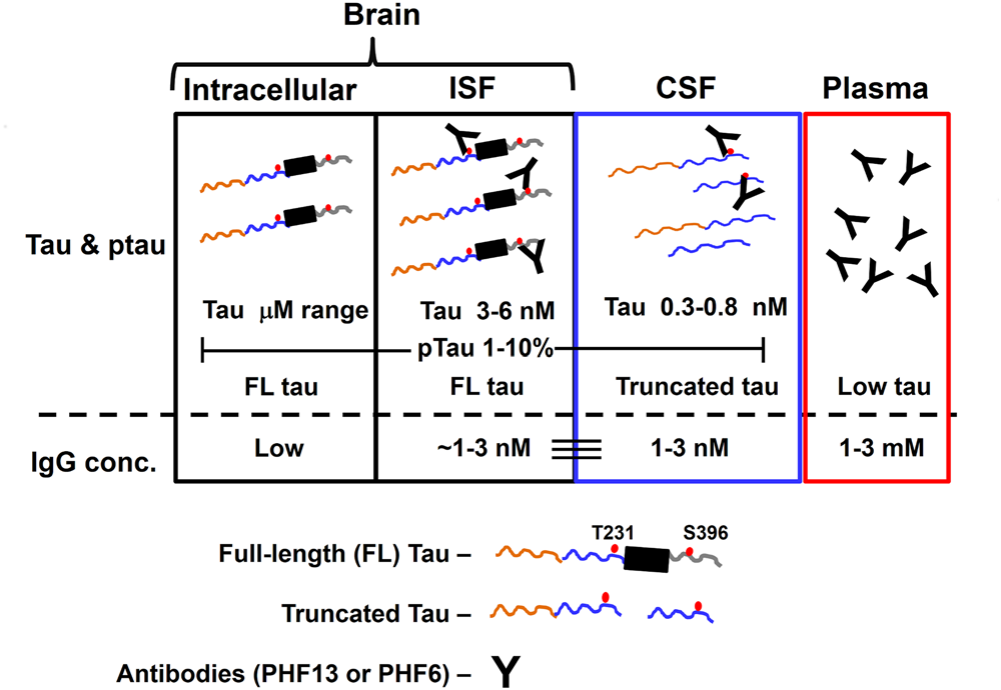
Model of antibody engagement of tau in-vivo. A compartmental model depicting tau and antibody (IgG) levels in brain, interstitial fluid (ISF), cerebrospinal fluid (CSF) and plasma. CSF tau is truncated with levels of ~1 nM, while tau in ISF exists as a full-length molecule with levels of 3–5 nM (4-5x greater than in CSF) [47]. Concentrations of p-tau are estimated to be about 1–10% of total tau levels. Tau antibody concentrations are 1–3 nM in CSF and ISF [44]. Antibody engagement of p-tau in CSF and ISF would enable clearance of tau via a variety of antibody-mediated mechanisms. Full-length tau is indicated as a molecule containing N-terminal (orange line), mid-domain (blue line), microtubule-binding repeat region (black box), and C-terminal (grey line) regions, whereas truncated tau in the CSF compartment is indicated by the mid-domain and N-terminal fragments.

We have previously reported that CSF tau exists predominantly as a truncated fragment that can be measured robustly with mid-domain and N-terminal tau assays, but is near the limit of detection when measured with antibodies recognizing C-terminal epitopes [37, 38]. Treatment with PHF13 reduces CSF tau and pT181 tau levels in rTg4510 mice, but binds to a C-terminal epitope (pS396) that is not detectable in CSF. In addition, it has been reported that ISF contains full-length tau [47, 48]. These findings suggest a potential model to explain the results obtained with the p-tau antibodies (Fig 8). We hypothesize that full- or nearly full-length tau is secreted from neurons into the interstitial fluid. The extracellular tau is likely hyperphosphorylated in aging rTg4510 mice, reflecting brain p-tau elevations (Fig 2). While traversing the ISF to the CSF, tau is cleaved into mid-domain and N-terminal fragments that are abundant in CSF. The fate of the C-terminal fragments resulting from such a scheme is unclear, but they could potentially be a “transmissible” species driving spread of tau pathology. Under such a scheme, the p-tau antibodies used in this study would sequester a fraction of the secreted tau and promote clearance, thereby reducing the p-tau and total tau fragments detectable in CSF. In summary, we believe that an antibody-mediated reduction of extracellular p-tau may partly drive CSF tau and p-tau reductions in rTg4510 mice.

A key question is whether the tau antibody exposures in our studies are sufficient to fully engage extracellular tau species in the CNS. In the PS19 tau model, ISF tau is in the range of 200–300 ng/mL (3–6 nM) while CSF tau levels were 20–40 ng/mL [47]. In rTg4510 mice, CSF total tau levels (using the mid-domain assays) were 20–40 ng/ml (0.3–0.8 nM) (this study and [37]). Since p-tau levels are estimated to be <10% of total tau (based on Fig 2A and 2B), we would expect reasonable engagement of extracellular p-tau at the low nM antibody exposures achieved in CSF. In particular, our findings of 20–40% reduction in brain p-tau and CSF tau levels with PHF6 and PHF13 are generally consistent with CSF antibody exposures of 1–3 nM and antibody K_D_ of 1–4 nM. CSF and ISF levels of antibody are ~0.1% of plasma levels, following systemic dosing (Table 2 and [44]. The fact that only a small fraction of soluble tau is phosphorylated may set a limit on the extent of soluble tau reduction observable with anti-p-tau antibodies. Taken together, the observed efficacy in primary neuronal and in vivo models with p-tau antibody treatment suggests that the toxic species of tau may be phosphorylated.

In addition to the studies in rTg4510 mice, the PHF13 antibody was also examined in a model in which tau pathology was induced by intracerebral injection of K18PL tau PFFs into young PS19 mice. In these young PS19 mice, the initial formation of tau pathology depends on the uptake of exogenous PFFs that then serve as templates for the recruitment and misfolding of the transgene-driven human tau and to a lesser extent endogenous mouse tau [10]. The time-dependent spread of tau pathology observed in this model is thought to result from a combination of intra-axonal transport of the injected PFFs from the sites of PFF injection, as well as similar transport of misfolded endogenous tau species via neural networks [10]. Administration of PHF13 to PFF-injected PS19 mice resulted in a significant reduction in tau pathology in the EC and improvement in a NOR cognitive assay. Moreover, there was evidence of reduced tau pathology in the contralateral hippocampus in a second large study utilizing this model. The contralateral hippocampus was examined because it is likely that the development of tau inclusions there depends, at least in part, on neuron-to-neuron transmission of misfolded tau. However, as there are bilateral CA3 projections [49], it is possible that some fraction of the observed contralateral hippocampal pathology results from direct axonal transport of tau PFFs. In fact, subtle differences in the site of K18PL PFF injection into the hippocampus may influence the proportion of tau pathology due to neuron-to-neuron spreading of PFF-induced tau pathology from the ipsilateral to contralateral hippocampus. In this regard, small disparities in the K18PL PFF injection coordinates may explain why PHF13 treatment reduced contralateral hippocampal tau pathology in studies conducted in one laboratory but not the other. Notwithstanding this discordant finding, the observation that PHF13 treatment did not affect insoluble tau pathology in the presumably cell autonomous rTg4510 model supports the idea that the reduction in tau pathology in the PS19 tau PFF-injection model is due, at least in part, to a slowing of pathological tau transmission. Overall, the results from the two distinct mouse models of tauopathy provide additional validation of tau antibody treatment as a potential therapeutic strategy for reducing pathologic tau and improving functional deficits with clinically achievable antibody exposures [43].

Key challenges still remain in terms of understanding the impact of anti-tau antibody treatment in tauopathy mouse models. Many published studies have focused on well characterized tau epitopes for active or passive immunization, including the pS396/pS404 epitope (PHF1), or have utilized conformational antibodies such as MC1 [25–29, 31]. One recent study compared both N-terminal and MTBR-binding antibodies via direct intracerebroventricular infusion (ICV) [30] in the PS19 mouse model. Although CSF antibody exposure was ~50 nM after ICV infusion (20-50x greater than IP dosing), relatively modest and variable effects were observed on brain soluble and insoluble tau, without clear relationships to behavioral endpoints [30]. Constraints on antibody diffusion from the ventricles to brain parenchyma may increase variability in antibody levels across brain regions and thus account in part for the modest effects in brain tau endpoints. Similar partial reductions of brain tau levels, as measured by biochemical or histological methods, are evident in both passive and active antibody treatment studies [25–30, 50]. Generally, in tau mouse models that are quite aggressive, with very high tau expression levels (4-10x) over endogenous mouse tau levels, it may be difficult to prevent or rescue pathology. However, a robust reduction of tau pathology has been reported in a tau mouse model [51] that expresses near physiological levels of tau [52]. The lack of robust reduction of brain tau in several of these studies may also be due to an inability of antibodies to impact intracellular tau, with primary effects on extracellular tau and cell-to-cell pathological transmission. Measurement of ISF tau before and after antibody treatment might provide a better understanding of antibody exposure and pharmacodynamic effects in animal models, but may still pose challenges related to the sensitivity required to detect low levels of ISF p-tau. Although tau pathology in human AD and tauopathies are similar in terms of an increase in tau NFT pathology, key differences exist in terms of the rate of disease progression, cell types affected by tau pathology and the site of initiation and spread of tau pathology, and the forms of tau that are present in the insoluble aggregates. Thus, further work is needed to elucidate the nature of the transmissible tau species in human neurodegenerative diseases and define the best epitope(s) for tau immunotherapy.

In conclusion, we have demonstrated that p-tau antibodies targeting pT231 and pS396 can reduce brain and CSF p-tau levels and improve function in aging tau transgenic mice. Moreover, these antibodies decrease tau pathology and improve behavior in a tau PFF-induced transmission model of tauopathy. These studies thus provide important insights regarding the potential of passive tau immunotherapy for the treatment of AD and related tauopathies.

## Supporting Information

S1 Fig Brain AT8 ELISA. **A.** Tg4510 mice brain soluble extracts show a linear dilution of signal in AT8 ELISA. Signal near background levels in Tau KO, t-TA (tetracycline transactivator) and DN (double transgene negative) mice. **B.** Tg4510 mice brain insoluble extracts show a linear dilution of signal in AT8 ELISA. Signal near background levels in Tau KO, tTA and DN mice. **C.** Brain AT8 tau signal is specific and not affected by Tau441, but competed with AT8 phospho-peptide (RSGYSSPGS-(PO4)PGT(PO4)PGSRSR) but not control AT8 peptide without phosphorylated sites at S-202 & T-205 (RSGYSSPGSPGTPGSRSR) or pT181 phospho-peptide (KTPPAPK-T(PO4)-PPSS). **D.** AT8 ELISA standard curve vs samples—brain soluble extracts. **E.** AT8 ELISA standard curve vs samples—brain insoluble extracts (from Fig 2).
(DOCX)

S2 Fig CSF pT181 Tau ELISA validation. We have previously reported on a robust human CSF pT181 tau ELISA assay [38]. **A.** In Tg4510 mice expressing human P301L tau, CSF samples showed robust linearity of signal with dilution. Dotted line indicates background signal. **B.** Tg4510 mice show specific pT181 tau signal with no interference from non-phosphorylated tau (Tau441) and competed by pT181 phospho-peptide (KTPPAPK-T(PO4)-PPSS). CSF pT181 tau signal was low in Tau knock-out mice (TauKO), Tau-tetracycline transactivator expressors (tTA), and double negative mice (DN). **C. D.** CSF total tau and pT181 tau signal from Tg4510 mice fell in the range of Tau441 and pT181 tau standard curves, respectively from (Fig 3).
(DOCX)

S3 Fig Brain AT8 immunostaining in Tg4510 mice from IgG2b, PHF6 and PHF13 treated animals. Images from 3 individual animals from each group are shown. No differences in staining was observed among the groups.
(DOCX)

S4 Fig Lack of direct interference of PHF13 or PHF6 antibodies in Total tau, AT8 ptau or pT181 tau ELISAs in Standard curves. PHF13 and PHF6 were spiked into samples at 0, 0.3, 3 and 10 ug/ml concentrations, respectively. Total Tau standard curves—Lack of interference with **A.** PHF13 and **B.** PHF6. AT8 ptau standard curves—Lack of interference with **C.** PHF13 and **D.** PHF6. pT181 tau standard curves—Lack of interference with **E.** PHF13 and **F.** PHF6.
(DOCX)

S5 Fig Lack of direct interference of PHF13 or PHF6 antibodies in Total tau, AT8 ptau or pT181 tau ELISAs in brain extracts. PHF13 and PHF6 were spiked into samples at 0, 0.3, 3 and 10 ug/ml concentrations, respectively. Brain total tau assay—Lack of interference with **A.** PHF13 and **B.** PHF6. Brain AT8 ptau assay——Lack of interference with **C.** PHF13 and **D.** PHF6.
(DOCX)

S6 Fig Lack of direct interference of PHF13 or PHF6 antibodies in Total tau and pT181 tau ELISAs in CSF. PHF13 and PHF6 were spiked into samples at 0, 0.3, 3 and 10 ug/ml concentrations, respectively. Human CSF was used as a surrogate due to limited availability of Tg4510 mice CSF samples. CSF total tau assay—Lack of interference with **A.** PHF13 and **B.** PHF6. CSF pT181 tau assay—Lack of interference with **C.** PHF13 and **D.** PHF6.
(DOCX)

S7 Fig Whole slide images and quantitation of brain sections stained with AT8. **A.** Regional distribution of tau pathology following PFF injection into hippocampus. Cartoon shows location of ipsilateral injection of PFF into cortex and hippocampus and spread to entorhinal cortex (EC) and locus ceruleus (LC). Top right panel—Images from 2 slides from all brain sections stained with AT8 from a single animal. Lower panel—Images from horizontal sections arranged from top to bottom of the brain to illustrate the distribution of AT8 staining. Red outlines drawn around EC region. Blue outlines around LC region. **B.** Lack of AT8 staining in PS19 mice injected intracranially with PBS. **C.** Image thresholding and segmentation of AT8 positive regions in hippocampus and EC.
(DOCX)

S8 Fig CSF total tau changes in PFF-injected PS19 mice. CSF total tau levels were evaluated in PS19 mice injected intracerebrally with PBS or K18PL PFFs. A small but significant increase in CSF tau was observed with PFF injection. A trend for reduction in CSF tau levels was observed with PHF13 when compared to IgG2b treated mice.
(DOCX)

S1 Table Gene expression changes in microglial and immune related markers. (PDF)
