# Supplementary material for: Passive Immunization with Phospho-Tau Antibodies Reduces Tau Pathology and Functional Deficits in Two Distinct Mouse Tauopathy Models
Source: PLoS One. 2015 May 1;10(5):e0125614. doi: 10.1371/journal.pone.0125614 (PMC4416899; doi:10.1371/journal.pone.0125614)
Supplement: S1 Fig — A. Tg4510 mice brain soluble extracts show a linear dilution of signal in AT8 ELISA. Signal near background levels in Tau KO, t-TA (tetracycline transactivator) and DN (double transgene negative) mice. B. Tg4510 mice brain insoluble extracts show a linear dilution of signal in AT8 ELISA. Signal near background levels in Tau KO, tTA and DN mice. C. Brain AT8 tau signal is specific and not affected by Tau441, but competed with AT8 phospho-peptide (RSGYSSPGS-(PO4)PGT(PO4)PGSRSR) but not control AT8 peptide without phosphorylated sites at S-202 & T-205 (RSGYSSPGSPGTPGSRSR) or pT181 phospho-peptide (KTPPAPK-T(PO4)-PPSS). D. AT8 ELISA standard curve vs samples—brain soluble extracts. E. AT8 ELISA standard curve vs samples—brain insoluble extracts (from Fig 2). (DOCX) [file pone.0125614.s001.docx]

**S1 Figure. Brain AT8 ELISA.**
