# Supplementary material for: Passive Immunization with Phospho-Tau Antibodies Reduces Tau Pathology and Functional Deficits in Two Distinct Mouse Tauopathy Models
Source: PLoS One. 2015 May 1;10(5):e0125614. doi: 10.1371/journal.pone.0125614 (PMC4416899; doi:10.1371/journal.pone.0125614)
Supplement: S2 Fig — We have previously reported on a robust human CSF pT181 tau ELISA assay [38]. A. In Tg4510 mice expressing human P301L tau, CSF samples showed robust linearity of signal with dilution. Dotted line indicates background signal. B. Tg4510 mice show specific pT181 tau signal with no interference from non-phosphorylated tau (Tau441) and competed by pT181 phospho-peptide (KTPPAPK-T(PO4)-PPSS). CSF pT181 tau signal was low in Tau knock-out mice (TauKO), Tau-tetracycline transactivator expressors (tTA), and double negative mice (DN). C. D. CSF total tau and pT181 tau signal from Tg4510 mice fell in the range of Tau441 and pT181 tau standard curves, respectively from (Fig 3). (DOCX) [file pone.0125614.s002.docx]

**S2 Figure. CSF pT181 Tau ELISA validation.**
