# Supplementary material for: Passive Immunization with Phospho-Tau Antibodies Reduces Tau Pathology and Functional Deficits in Two Distinct Mouse Tauopathy Models
Source: PLoS One. 2015 May 1;10(5):e0125614. doi: 10.1371/journal.pone.0125614 (PMC4416899; doi:10.1371/journal.pone.0125614)
Supplement: S3 Fig — Images from 3 individual animals from each group are shown. No differences in staining was observed among the groups. (DOCX) [file pone.0125614.s003.docx]

**S3 Figure. Brain AT8 immunostaining in Tg4510 mice treated with IgG2b, PHF6, and PHF13.**
