# Supplementary material for: Passive Immunization with Phospho-Tau Antibodies Reduces Tau Pathology and Functional Deficits in Two Distinct Mouse Tauopathy Models
Source: PLoS One. 2015 May 1;10(5):e0125614. doi: 10.1371/journal.pone.0125614 (PMC4416899; doi:10.1371/journal.pone.0125614)
Supplement: S4 Fig — PHF13 and PHF6 were spiked into samples at 0, 0.3, 3 and 10 ug/ml concentrations, respectively. Total Tau standard curves—Lack of interference with A. PHF13 and B. PHF6. AT8 ptau standard curves—Lack of interference with C. PHF13 and D. PHF6. pT181 tau standard curves—Lack of interference with E. PHF13 and F. PHF6. (DOCX) [file pone.0125614.s004.docx]

**S4 Figure. Lack of direct interference of PHF13 or PHF6 antibodies in Total tau, AT8 ptau or pT181 tau ELISA assays in Standard curves.**
