# Supplementary material for: Passive Immunization with Phospho-Tau Antibodies Reduces Tau Pathology and Functional Deficits in Two Distinct Mouse Tauopathy Models
Source: PLoS One. 2015 May 1;10(5):e0125614. doi: 10.1371/journal.pone.0125614 (PMC4416899; doi:10.1371/journal.pone.0125614)
Supplement: S5 Fig — PHF13 and PHF6 were spiked into samples at 0, 0.3, 3 and 10 ug/ml concentrations, respectively. Brain total tau assay—Lack of interference with A. PHF13 and B. PHF6. Brain AT8 ptau assay——Lack of interference with C. PHF13 and D. PHF6. (DOCX) [file pone.0125614.s005.docx]

**S5 Figure. Lack of direct interference of PHF13 or PHF6 antibodies in Total tau and AT8 ptau ELISA assays in brain extracts.**
