# Supplementary material for: Passive Immunization with Phospho-Tau Antibodies Reduces Tau Pathology and Functional Deficits in Two Distinct Mouse Tauopathy Models
Source: PLoS One. 2015 May 1;10(5):e0125614. doi: 10.1371/journal.pone.0125614 (PMC4416899; doi:10.1371/journal.pone.0125614)
Supplement: S6 Fig — PHF13 and PHF6 were spiked into samples at 0, 0.3, 3 and 10 ug/ml concentrations, respectively. Human CSF was used as a surrogate due to limited availability of Tg4510 mice CSF samples. CSF total tau assay—Lack of interference with A. PHF13 and B. PHF6. CSF pT181 tau assay—Lack of interference with C. PHF13 and D. PHF6. (DOCX) [file pone.0125614.s006.docx]

**S6 Figure. Lack of direct interference of PHF13 or PHF6 antibodies in Total tau and pT181 tau ELISA assays in CSF.**  Antibody interference experiments were done using human CSF, due to limited amount of sample available from mice (~10 µL/mouse). Human CSF has previously been demonstrated to have robust signal in both the total tau and pT181 tau assays (Meredith et al 2013).
