# Supplementary material for: Passive Immunization with Phospho-Tau Antibodies Reduces Tau Pathology and Functional Deficits in Two Distinct Mouse Tauopathy Models
Source: PLoS One. 2015 May 1;10(5):e0125614. doi: 10.1371/journal.pone.0125614 (PMC4416899; doi:10.1371/journal.pone.0125614)
Supplement: S7 Fig — A. Regional distribution of tau pathology following PFF injection into hippocampus. Cartoon shows location of ipsilateral injection of PFF into cortex and hippocampus and spread to entorhinal cortex (EC) and locus ceruleus (LC). Top right panel—Images from 2 slides from all brain sections stained with AT8 from a single animal. Lower panel—Images from horizontal sections arranged from top to bottom of the brain to illustrate the distribution of AT8 staining. Red outlines drawn around EC region. Blue outlines around LC region. B. Lack of AT8 staining in PS19 mice injected intracranially with PBS. C. Image thresholding and segmentation of AT8 positive regions in hippocampus and EC. (DOCX) [file pone.0125614.s007.docx]

**S7 Figure. Whole-slide images and quantitation of brain sections stained with AT8**
