# Supplementary material for: Passive Immunization with Phospho-Tau Antibodies Reduces Tau Pathology and Functional Deficits in Two Distinct Mouse Tauopathy Models
Source: PLoS One. 2015 May 1;10(5):e0125614. doi: 10.1371/journal.pone.0125614 (PMC4416899; doi:10.1371/journal.pone.0125614)
Supplement: S8 Fig — CSF total tau levels were evaluated in PS19 mice injected intracerebrally with PBS or K18PL PFFs. A small but significant increase in CSF tau was observed with PFF injection. A trend for reduction in CSF tau levels was observed with PHF13 when compared to IgG2b treated mice. (DOCX) [file pone.0125614.s008.docx]

**S8 Figure. CSF total tau changes in PFF-injected PS19 mice**
