# Supplementary material for: Passive Immunization with Phospho-Tau Antibodies Reduces Tau Pathology and Functional Deficits in Two Distinct Mouse Tauopathy Models
Source: PLoS One. 2015 May 1;10(5):e0125614. doi: 10.1371/journal.pone.0125614 (PMC4416899; doi:10.1371/journal.pone.0125614)
Supplement: S1 Table — (PDF) [file pone.0125614.s009.pdf]

S1 Table. Gene expression changes in microglial and immune related markers

Gene ID.	Tg4510 vs DN	Age	Treatment
**M1 (Classical)**			
TNFa	Yes	Yes	No
Il12a	No	No	No
Nos2	No	No	No
**M2**			
IL10	NA	NA	NA
Arg1	No	No	No
TGF beta	No	No	No
**Fc gamma (M2b)**			
CD64	Yes	Yes	No
CD32	Yes	Yes	No
CD16	Yes	Yes	No
TREM2	Yes	Yes	No
CD33, Siglec	Yes	Yes	No
**Complement**			
C3	Yes	Yes	No
C4	Yes	Yes	No
**General**			
GFAP	Yes	Yes	No
CD14	Yes	Yes	No
CD68	Yes	Yes	No

NA - below assay sensitivity

Genes showing significant increases in expression based on genotype – Tg4510 vs DN mice, with age – 3 vs 6 month old Tg4510 mice. No significant changes observed with PHF13 & PHF6 antibody treatment from 3 to 6 months of age.

RT-PCR assays to evaluate inflammation and immune related endpoints - RNA was prepared from frontal cortex using RNAeasy Plus (Qiagen). cDNA was generated by reverse transcribing 300 ng of cortical or hippocampal RNA using the High Capacity RNA-to-cDNA Kit (Applied Biosystems, Carlsbad, CA) according to the manufacturer‘s protocol. qPCR was conducted in triplicate for each target gene and each sample on a 7900 Fast Real-Time PCR System (Applied Biosystems, Carlsbad, CA) using the following cycling parameters: 1x 95°C 10 minutes, 40x 95°C 25 seconds, 60°C 1 minute. Target genes that were evaluated included M1 family - TNFa, Il12a, Nos2; M2 family - IL10, Arg1,TGF beta, Fc gamma (M2b), CD64, CD32, CD16, TREM2, CD33, Siglec; Complement members - C3, C4, and other general astrocytic and microglial markers – GFAP, CD14, and CD68. Validated

mouse primer sets using Taqman® Gene Expression Assays were employed to evaluate the above genes (Invitrogen, CA). Gene expression levels were determined using the ΔΔCt relative quantification method. Specifically, the technical replicates of the cycle threshold (Ct) values were averaged for each sample and the average Ct for the reference gene (Δ-actin) was subtracted from the average Ct of the target gene to give the ΔCt value for each sample. Coefficients of variation ranged from 0.00089-1.56% for technical replicates, and 6.05-23.86% for biological replicates. To calculate the ΔΔCt, the average ΔCt of the vehicle-treated animals was subtracted from the average ΔCt of the experimental sample. To calculate relative expression, the formula $2^{-\Delta\Delta Ct}$ was used, which assumes doubling of the amplicon every amplification cycle.

Taqman Gene expression assays (Invitrogen)

**Gene ID.**	**Catalog ID.**	**Fluorophore**
Arg1	Mm00475988_m1	FAM
C3	Mm00437838_m1	VIC
C4	Mm00437890_m1	VIC
CD14	Mm00438094_g1	FAM
CD16	Mm00438882_m1	FAM
CD32	Mm00438875_m1	VIC
CD33	Mm00491152_m1	VIC
CD64	Mm00438874_m1	FAM
CD68	Mm03047340_m1	FAM
GAPDH	Mm99999915_g1	FAM
GFAP	Mm01253033_m1	FAM
IL10	Mm00439614_m1	FAM
Il12a	Mm00434165_m1	FAM
Nos2	Mm00440502_m1	VIC
TGFbeta	Mm01298616_m1	VIC
TNFalpha	Mm00443260_g1	FAM
TREM2	Mm04209424_g1	FAM
hTau	Hs00902193_m1	FAM
Eno2	Mm00469062_m1	VIC
